# The influenza virus RNA polymerase as an innate immune agonist and antagonist

**DOI:** 10.1007/s00018-021-03957-w

**Published:** 2021-10-22

**Authors:** Elizaveta Elshina, Aartjan J. W. te Velthuis

**Affiliations:** 1 Division of Virology, Department of Pathology, University of Cambridge, Addenbrooke’s Hospital, Hills Road, Cambridge, CB2 0QQ UK; 2 Lewis Thomas Laboratory, Department of Molecular Biology, Princeton University, Washington Road, Princeton, 08544 NJ USA

**Keywords:** RIG-I, mvRNA, DVG, RdRp, Innate immune response, PB1, PB2, PA, IFN

## Abstract

Influenza A viruses cause a mild-to-severe respiratory disease that affects millions of people each year. One of the many determinants of disease outcome is the innate immune response to the viral infection. While antiviral responses are essential for viral clearance, excessive innate immune activation promotes lung damage and disease. The influenza A virus RNA polymerase is one of viral proteins that affect innate immune activation during infection, but the mechanisms behind this activity are not well understood. In this review, we discuss how the viral RNA polymerase can both activate and suppress innate immune responses by either producing immunostimulatory RNA species or directly targeting the components of the innate immune signalling pathway, respectively. Furthermore, we provide a comprehensive overview of the polymerase residues, and their mutations, associated with changes in innate immune activation, and discuss their putative effects on polymerase function based on recent advances in our understanding of the influenza A virus RNA polymerase structure.

**Supplementary Information:**

The online version contains supplementary material available at 10.1007/s00018-021-03957-w.

## Introduction

A 100 years after the devastating influenza pandemic of 1918, influenza A viruses continue to pose a serious threat to human health. They infect up to twenty percent of the global population each year, resulting in several hundred thousand deaths [1]. In addition, influenza A viruses have a well-established zoonotic potential and ability to cause pandemics in naïve populations. In humans, influenza disease typically manifests itself as a mild-to-severe respiratory disease, but influenza A virus infections can occasionally spread beyond the respiratory tract. In humans and animal models, viral antigens have also been detected in the nervous system and heart [2–4].

Many factors contribute to influenza disease severity, including underlying risk factors, bacterial co-infections, and the innate immune response to infection [5, 6]. During an influenza virus infection, the innate immune response is typically activated when viral RNA (vRNA) is detected by the cytoplasmic or nuclear retinoic acid-inducible gene I (RIG-I) [7–9]. Subsequent signalling events lead to the production of interferons and other cytokines, which together mount a robust antiviral defence, and attract leukocytes and lymphocytes to clear the IAV infection. Counterintuitively, the virus can benefit from the pro-inflammatory responses in the lung, if it can use recruited leukocytes as additional targets for replication, as was shown for a low-dose influenza A virus infection [10]. Another outcome of infection is a disproportional and prolonged innate immune activation, commonly referred to as ‘cytokine storm’, that can cause tissue damage, acute lung injury, or severe acute respiratory distress syndrome [11, 12]. Prolonged exposure to IFNs, which are one group of cytokines overproduced during the ‘cytokine storm’, has also been linked to impaired lung repair and an increased susceptibility to bacterial infection after influenza virus infection in mice [13]. The cytokine storm is particularly common in infections with highly virulent influenza A virus strains, such as highly pathogenic avian H5N1 and H7N9 viruses, and the 1918 pandemic H1N1 strain, which trigger an overproduction of interferons and pro-inflammatory cytokines in the lower respiratory tract [14–17]. Several viral and host determinants of the immune dysregulation have been proposed, but the molecular mechanisms that allow these factors to start a cytokine storm are still poorly understood (reviewed in [18]).

**Fig. 1:**
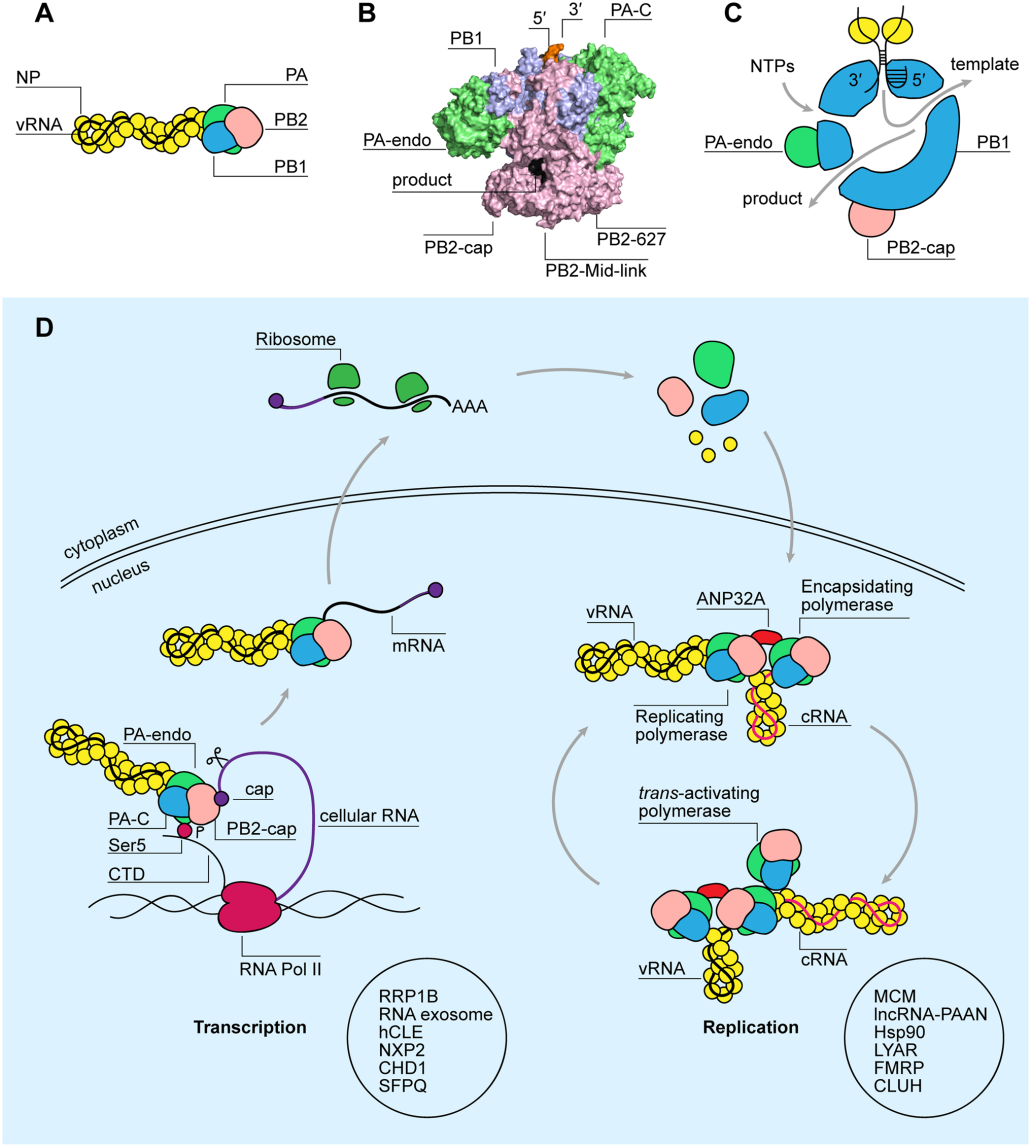
Structure and function of the influenza A virus RNA polymerase. **A** A schematic representation of an RNP, in which both termini of the vRNA are bound by the viral polymerase and the rest is associated with NP. **B** A surface representation of the human H3N2 influenza A virus RNA polymerase (PDB 6RR7) bound to the 3′ and 5′ promoter and the capped RNA (black; labelled as product). PA C-terminal (PA-C) and endonuclease (PA-endo) domains and PB2 cap-binding (PB2-cap), Mid-link and 627 domains are indicated. **C** A model of influenza A virus polymerase showing the active site cavity within the PB1 subunit. The location of the four channels that lead to the active site and the binding pockets of the 3′ and 5′ RNA termini are indicated. **D** Schematic representation of influenza virus genome transcription and replication. During transcription PA-C interacts with the Ser5-phosphorylated CTD of a transcribing RNA Pol II. PB2-cap binds the cap structure of the nascent cellular RNA, which is subsequently cleaved by PA and used to prime viral mRNA synthesis. Capped and polyadenylated viral mRNAs are translated in the cytoplasm by the host ribosomes. The newly made components of the viral RNP are transported into the nucleus by importins where they promote viral replication. During cRNA synthesis from a vRNA template, a newly made encapsidating polymerase forms a dimer with a replicating polymerase, while ANP32A acts as a bridge between the polymerases. During vRNA synthesis from a cRNA template a newly made trans-activating polymerase forms a dimer with the replicating polymerase, stimulating replication. An encapsidating-replicating polymerase dimer is also likely to form during vRNA synthesis with the aid of ANP32A to promote vRNP assembly. Examples of other cellular factors that are involved in either transcription or replication are shown at the bottom of the diagram

Activation of the innate immune response is triggered by the replication of the viral genome [19]. The influenza A virus genome consists of eight segments of negative-sense single-stranded vRNA that vary in length from 890 to 2341 nucleotides and code for ten major proteins, such as nucleoprotein (NP), non-structural protein 1 (NS1), and polymerase subunits [polymerase acidic (PA), polymerase basic 1 and 2 (PB1 and PB2)], as well as various accessory proteins, such as PA-X and PB1-F2 [20]. Each vRNA segment is encapsidated by a double-helical filament of NP and capped by a copy of the viral RNA polymerase, forming a viral ribonucleoprotein (vRNP) (Fig. 1A). It is in the context of these RNPs that the viral RNA polymerase copies and transcribes the genome segments [21]. In addition to making full-length copies of the viral genes, the viral RNA polymerase produces various aberrant RNA products that contain deletions in the genome segments, such as defective viral genomes (DVGs), mini viral RNAs (mvRNAs) and small viral RNAs (svRNAs) [22]. Various lines of research suggest that DVGs and mvRNAs play a role in activating the innate immune response through RIG-I [23, 24].

Several viral proteins, such as NS1, PA-X and PB1-F2, have well-established functions as innate immune modulators [25, 26]. However, these proteins do not explain the full complexity of the activation and inhibition of the innate immune response during infection. A factor, whose role in triggering and antagonising the innate immune response has been less comprehensively analysed, is the influenza A virus RNA polymerase. The role of the influenza virus RNA polymerase in innate immune responses to infection became evident in genome segment reassortment studies, in which polymerase encoding segments were exchanged between different virus strains or isolates. Together those studies demonstrated that the polymerase genes not only significantly affect virulence and innate immune signalling, but also likely contribute to cytokine dysregulation during infection with highly pathogenic virus strains [27–31] (full list in Supplementary Table 1). Similarly, innate immune activation can be induced by single mutations in the polymerase genes or combinations of mutations in one or more genes [24, 32, 33] (full list in Supplementary Table 2). Additional studies have shown that elimination of the interferon pressure during infection leads to substitution of conserved polymerase amino acids, indicating that the RNA polymerase amino acid composition is under a selection pressure from the immune response [34, 35].

In the last decade, significant advances have been made in understanding influenza A virus genetics, vRNA and vRNP structure, and the catalytic activity of the influenza A virus RNA polymerase [21]. However, the mechanisms underlying the immuno-stimulatory and -inhibitory effects of the viral polymerase are complex and diverse, and they have, to our best knowledge, not been comprehensively reviewed in light of these recent advances. Here, we aim to bring together our current understanding of the RNA polymerase, the role of the RNA polymerase in the innate immune response during influenza virus infection, and the polymerase mutations that affect innate immune signalling and host adaptation. By framing these concepts within our understanding of the RNA polymerase structure, we hope to extend our knowledge of the innate immune activation during influenza virus infection and the outcome of influenza disease.

## Structure of the viral RNA polymerase

The viral RNA polymerase transcribes and replicates the vRNA segments in the context of vRNPs (Fig. 1A). Within an RNP, the RNA polymerase binds to the viral promoter, which is formed by partially complementary 5′ and 3′ termini of the bound vRNA segment [36–38]. This organisation ensures that the vRNA termini as well as the RNA polymerase reside at one end of the RNP.

The influenza virus RNA polymerase is comprised of three subunits: PB1, PB2 and PA (Fig. 1B). The PB1 subunit contributes most to the RNA polymerase core and contains a typical RNA-dependent RNA polymerase (RdRp) domain fold, with fingers, thumb and palm subdomains. The three subdomains give rise to the structure of the active site and provide the residues that coordinate nucleotide incorporation [39–41]. The carboxy-terminal domain of PA and the amino-terminal third of PB2 also contribute to the RNA polymerase core, and in particular the thumb subdomain of the active site. The active site can be accessed by four channels that direct the movement of the RNA template in and out of the active site, the RNA product out of the active site, and nucleotides towards the active site (Fig. 1C). The RNA polymerase core is surrounded by several additional domains that are attached to linkers and support the transcriptional activity of the RNA polymerase [40–42]. These additional domains include an endonuclease domain that resides in the amino-terminal third of PA and a cap-binding domain that resides in the C-terminal third of PB2. Other important PB2 domains include the N-terminal domain, Mid-link, 627 domain, and nuclear localisation signal (NLS) domain [40].

## Function of the viral RNA polymerase

Upon viral entry and release of the vRNPs from the virion into the cytoplasm, the vRNPs are transported by host cell importins into the nucleus where primary transcription takes place (Fig. 1D) [43]. The process of viral transcription is initiated when the PA C-terminus of the incoming vRNP associates with the C-terminal domain (CTD) of an initiating, serine-5 phosphorylated cellular RNA polymerase II (Pol II). Following this interaction, the cap-binding domain of PB2 binds the cap-structures of nascent Pol II RNAs, ensuring that they can be cleaved by the endonuclease domain of PA. The resulting capped primers are 10–14 nucleotides long and used by PB1 as primers for viral transcription [41, 44, 45]. Viral transcription terminates when the viral RNA polymerase stutters on a poly-uridine track at the 5′ terminus of the vRNA template, producing a polyA tail [41, 46]. The resulting viral mRNAs are translated by host ribosomes into new viral proteins required for viral replication (Fig. 1D). These new viral proteins are transported from the cytoplasm to the nucleus by host cell importins [47].

In contrast to viral transcription, viral replication is a two-step process that starts with the generation of a complementary RNA (cRNA) intermediate using a primer-independent initiation mechanism (Fig. 1D). As the nascent cRNA leaves the active site through the product exit channel, it must be encapsidated by a newly synthesized RNA polymerase and nucleoproteins [21, 22]. To start the encapsidation process, the replicating polymerase and new polymerase must form a dimer that is stabilised by host protein Acidic Nuclear Phosphoprotein 32 Family Member A (ANP32A) [48–54]. Next, the new RNA polymerase in the resulting cRNP uses the cRNA as template for vRNA synthesis [55]. However, the initiation of vRNA synthesis requires a trans-activating or regulatory polymerase, as well as an encapsidating RNA polymerase and ANP32A. The trans-activating polymerase and the replicating polymerase form a dimer that is distinct from the encapsidating-replicating polymerase dimer (Fig. 1D). This dimer is likely formed to ensure that enough newly made viral proteins are available to assemble the nascent vRNA into a vRNP, and to trigger an essential realignment step during the initiation of vRNA synthesis when this condition is met [48, 56, 57].

Besides Pol II and ANP32A, viral transcription and replication are assisted by a number of other cellular proteins, many of which also directly interact with the viral polymerase (reviewed in [58]). These host factors participate in different stages of RNA synthesis, such as cap snatching (rRNA processing 1 homolog B or RRP1B, RNA exosome), general transcription (chromodomain helicase DNA-binding 1 or CHD1, hCLE and NXP2) polyadenylation (splicing factor proline-glutamine rich or SFPQ), cRNA synthesis (minichromosome maintenance or MCM) nuclear import and assembly of the polymerase (importins, long non-coding RNA-PAAN or lncRNA-PAAN, heat shock protein 90 or Hsp90), and vRNP assembly and transport (LYAR, FMRP and CLUH) (Fig. 1D) [58].

## RIG-I signalling pathway activation by IAV

Pathogen receptors, or pattern recognition receptors (PRRs), start the innate immune response during an infection. PRRs function by binding to conserved structures, or pathogen-associated molecular patterns (PAMPs), and activating signalling cascades that trigger expression of innate immune genes, such as type I and type III interferons. There are at least three PRR protein families involved in the recognition of an influenza virus infection, including toll-like receptors (TLRs), the nucleotide oligomerization domain (NOD)-like receptors (NLRs) and RIG-I-like receptors (RLRs) [59]. In addition, influenza A virus RNA is bound by Z-DNA binding protein 1, an activator of necroptosis [60].

**Fig. 2:**
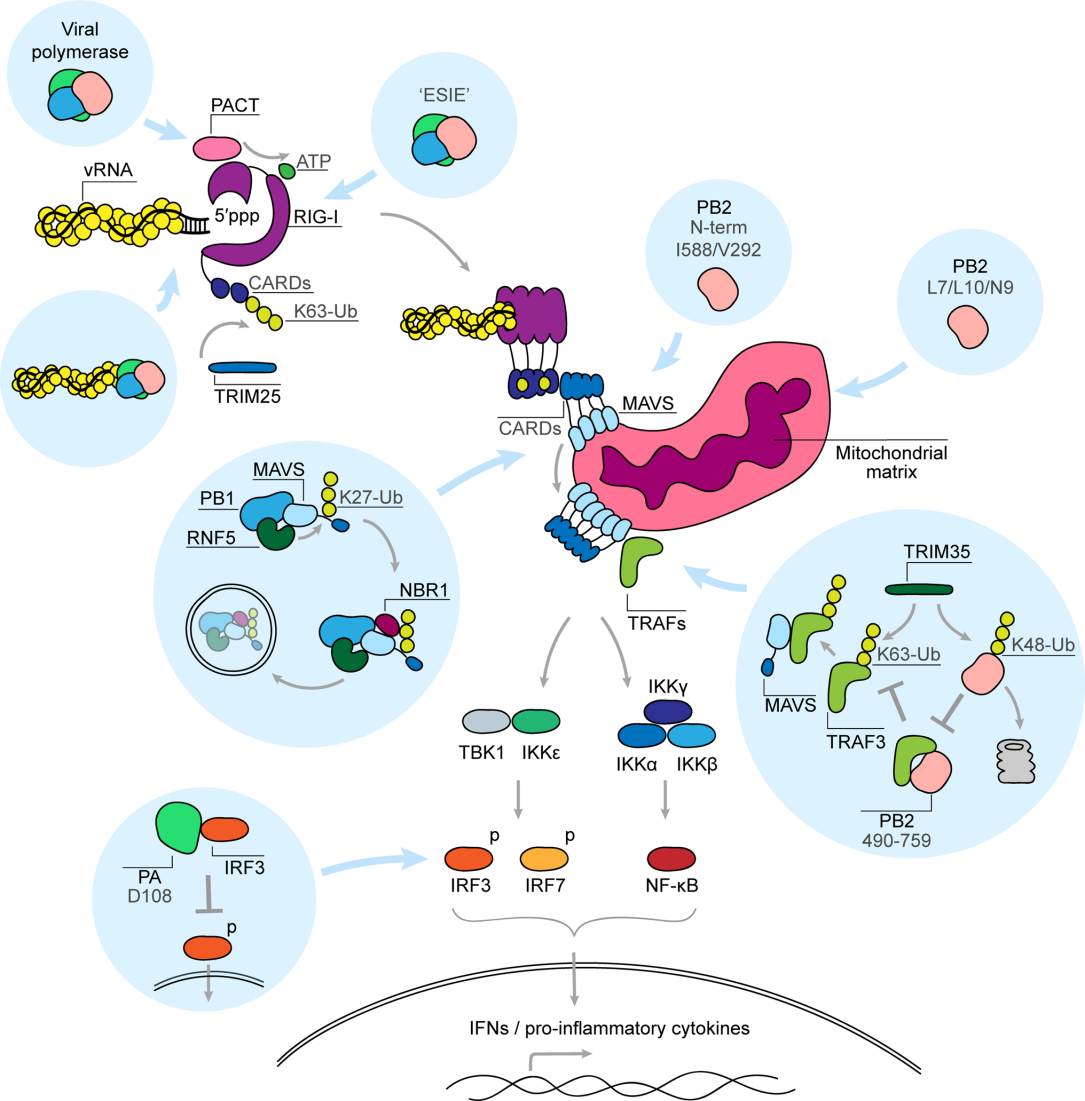
RIG-I signalling pathway and its interaction with the influenza A virus polymerase subunits. Centre left to centre bottom: the RIG-I signalling pathway is activated upon binding of an RNA ligand, such as the 5′ppp-dsRNA region of the influenza virus promoter, by the CTD of RIG-I. A subsequent ATP-dependent conformational change exposes two N-terminal CARDs of RIG-I and promotes RIG-I translocation along the RNA and formation of a RIG-I oligomer. One of the host proteins that potentiates RIG-I signalling is PKR activating protein (PACT), which binds to the CTD domain of RIG-I and enhances ATPase activity. RIG-I filament formation brings RIG-I CARDs into close proximity, facilitating formation of a CARD tetramer. The tetramer is stabilised by ubiquitin chains that are added by TRIM25. The RIG-I complex then migrates towards the mitochondrial outer membrane, where it associates with MAVS. The interaction between the CARD domains of RIG-I and MAVS nucleates MAVS filament formation. MAVS aggregation induces binding of TRAF family E3 ubiquitin ligases to MAVS, which potentiate recruitment of IKKs. Activated IKKε and TBK1 phosphorylate transcriptional factors IRF3 and IRF7, while transcriptional factor NF-κB is activated by the IKKα/β/γ complex. The activated IRF3, IRF7 and NF-κB translocate into the nucleus where they induce transcriptional activation of IFN and pro-inflammatory cytokine genes. Top left: the influenza virus RNA polymerase shields viral promoter from RIG-I recognition. Polymerase subunits also bind RIG-I in an ‘ESIE’-motif-dependent manner and interact with PACT. Middle: PB2 binding to MAVS is associated with inhibition of IFN expression and is attributed either to its N-terminal domain or to PB2 amino acids 588 and 292. PB1 induces degradation of MAVS by forming a complex with MAVS, RNF5 and NBR1. PB1 induces Lys27-linked ubiquitination of MAVS by RNF5, which is then recognised by NBR1 that targets ubiquitinated MAVS for autophagic degradation. Bottom right: PB2 (residues 490–759) binds to TRAF3 and prevents its Lys63-linked polyubiquitination by TRIM35, disrupting the formation of TRAF3-MAVS complex and preventing activation of TBK1 and IKKε kinases. TRIM35, in turn, induces Lys48-linked polyubiquitination of PB2, targeting it for proteasomal degradation. PB2 is also targeted to mitochondria via residues L7, L10 and N9. Bottom left: PA inhibits IFN production by binding to IRF3 and precluding its phosphorylation and nuclear translocation

In most cell types, the RIG-I signalling pathway (Fig. 2) plays a key role in detecting influenza A virus RNA [8, 9, 61]. Only in plasmocytoid dendritic cells are the influenza virus RNA molecules mainly detected by the endosomal TLR7 [62, 63]. RIG-I is activated when its C-terminal domain (CTD) binds the partially double-stranded 3′ and 5′ termini of the vRNA or cRNA promoter, so-called ‘panhandle’ [7, 64, 65] (Fig. 2). This binding leads to an ATP-dependent conformational change in RIG-I that exposes the N-terminal caspase activation and recruitment domains (CARDs) [66]. At the same time, the RNA-binding domains of RIG-I (CTD and helicase) translocate along the RNA ligand, bringing several RIG-I molecules and their CARDs into proximity [67–69]. The exposed CARDs of RIG-I are next polyubiquitinated by the tripartite motif-containing protein 25 (TRIM25), which promotes formation of CARD tetramers [70, 71]. CARD tetramers of RIG-I subsequently bind to the CARDs of mitochondrial antiviral-signalling protein (MAVS), nucleating MAVS filament formation—a step necessary for subsequent signal transduction [72]. MAVS oligomerisation leads to recruitment of downstream signalling molecules, such as TNF receptor-associated factor (TRAF) family E3 ubiquitin ligases and inhibitor of NF-κB kinase (IKK) family members. IKKs subsequently activate interferon-regulatory factors 3 and 7 (IRF3, IRF7) and NF-κB, which translocate into the nucleus to promote the transcription of interferon and pro-inflammatory cytokine genes [73].

**Fig. 3:**
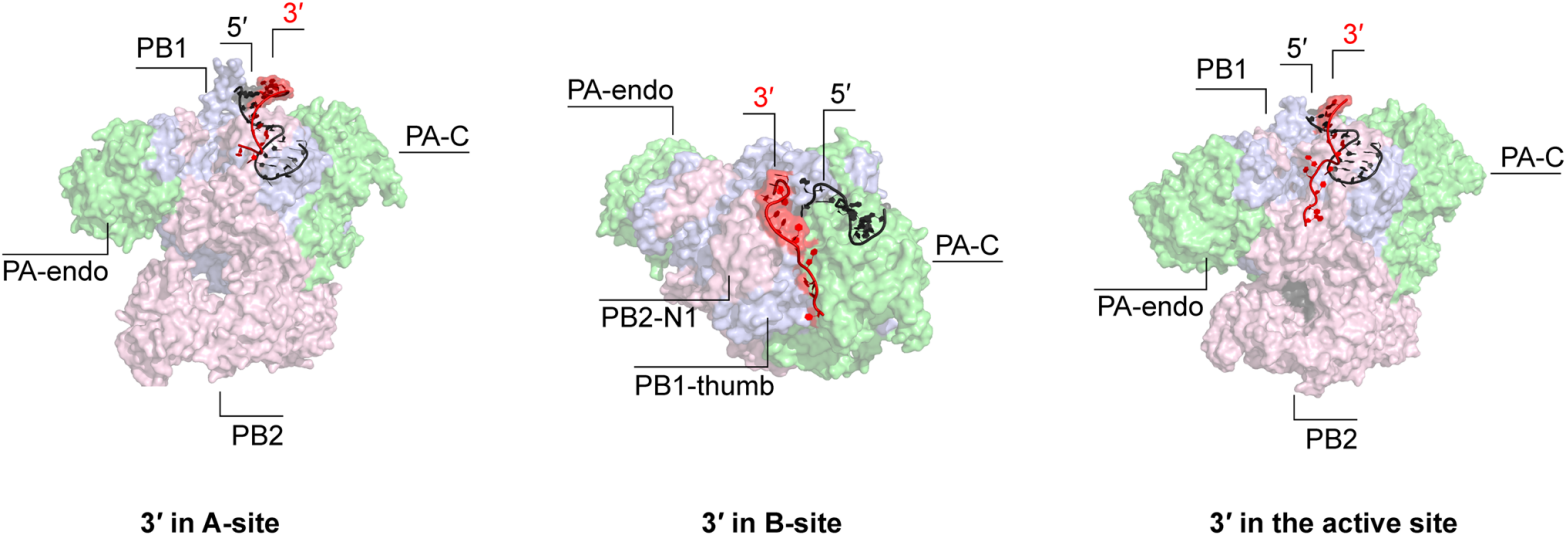
The conformations of the viral promoter when bound to the polymerase heterotrimer. Left: bat H17N10 influenza A polymerase with bound vRNA promoter (PDB 4WSB). Middle: bat H17N10 influenza A polymerase pre-termination complex with 3′ terminus threaded through the template exit channel and bound at the polymerase surface (top view) (PDB 6SZU). Right: human H3N2 influenza A virus polymerase bound to a vRNA promoter and a capped primer with 3′ terminus entering the active site (PDB 6RR7). Viral RNA polymerases are shown in surface representation and the viral promoter strands as a cartoon

Our understanding of the mechanisms behind the interactions between RIG-I and the viral promoter are complicated by the fact that the viral RNA polymerase shields the partially double-stranded promoter from RIG-I recognition in the context of an RNP. The RNA polymerase binds the first 10 residues of the 5′ terminus in a hook structure within a binding pocket consisting of PA and PB1 residues. The 3′ terminus, on the other hand, exists in at least three positions: either bound above the active site (A-site), on the outside of the PB2-N1 and PB1 thumb subdomain (B-site), or in the active site (Fig. 3) [36, 41, 56, 74]. Even during viral genome replication, nascent RNA is likely directly encapsidated by a new polymerase [48, 55]. Nevertheless, base-pairing of the terminal promoter region, which occurs in the absence of polymerase, is important for RIG-I activation [7]. It has been proposed that influenza viruses evolved a promoter that is not completely double-stranded, such as observed in other negative-strand RNA viruses, and that interruptions in the duplex reduce RIG-I activation relative to a fully base-paired promoter [7, 75]. It, therefore, remains unknown at which stage of the viral life cycle RIG-I is able to gain access to the viral promoter and initiate signalling.

## Interference of the viral RNA polymerase with the components of RIG-I signalling pathway

Besides its major role in genome replication and transcription, the influenza A virus polymerase and its individual subunits specifically interact with and inhibit several components of the RIG-I signalling pathway (Fig. 2).

### Viral polymerase binds RIG-I and its adaptor protein PACT

Several studies have demonstrated a direct interaction between the RNA polymerase or the vRNPs and RIG-I [32, 76, 77]. Weber et al. [76] showed that incoming vRNPs with an avian-adapted 627E residue in PB2 were more efficiently bound by RIG-I than those with the mammalian-adapted 627K signature. However, this difference in binding did not affect innate immune signalling. Instead, the authors proposed that the interaction allows RIG-I to block replication of 627E-containing viruses in mammalian cells [76]. Similarly, Li et al. [77] showed that all three polymerase subunits of a H9N2 virus strain bind RIG-I, and that this interaction does not result in innate immune activation. By contrast, Liedmann et al. [32, 78] identified an ‘ESIE’ motif consisting of PB1 (398E/524S/563I) and PA (351E) residues, which not only enhances the binding of the viral RNA polymerase to RIG-I, but also inhibits innate immune activation when compared to the ‘GGRK’ variant of the same motif (Fig. 2). Except PB1 398E, the motif’s residues are conserved and located on the thumb subdomain side of the RNA polymerase. By contrast, PB1 398E is localised at the opposite side of the RNA polymerase, and it is therefore unknown how these residues collectively contribute to RIG-I binding and whether separate residues might exhibit different immunomodulatory effects.

All three subunits of the viral RNA polymerase also interact with the PKR activating protein (PACT), an activator of PKR and RIG-I [79, 80]. In the case of RIG-I, PACT binds to the RIG-I CTD and triggers ATPase activity [80]. Chan et al. [81] showed that overexpression of the polymerase subunits diminishes IFN-β promoter activation during overexpression of RIG-I and PACT in the absence of vRNA. Additional experiments showed that the overexpressed polymerase subunits can co-precipitate with PACT, in the absence of vRNA, and that knockdown of endogenous PACT stimulates influenza A virus polymerase activity. The interaction between the RNA polymerase and PACT can be interpreted as a viral strategy to interfere with host innate immune signalling as well as an antiviral strategy of the host cell [81]. At present, it is unclear whether the observed immunomodulatory effects are directly linked to the interaction of the viral polymerase with PACT or that they derive from a reduced activation of RIG-I, or both.

### Polymerase subunits target MAVS, TRAF3 and IRF3

The PB1 subunit of the RNA polymerase was recently found to inhibit RIG-I signalling by inducing autophagic degradation of MAVS [82]. Specifically, PB1 forms a complex with MAVS and E3 ligase RNF5. This complex allows RNF5 to add Lys27-linked ubiquitin to MAVS (Fig. 2), which is recognised by an autophagic receptor, neighbour BRCA1 (NBR1). NBR1 recognition subsequently targets MAVS for autophagic degradation, inhibiting MAVS-mediated signalling [82].

The PB2 subunit of the RNA polymerase also inhibits innate immune signalling by targeting MAVS [83, 84]. The MAVS-interacting region of PB2 was mapped to the last 37 residues of its N-terminus, but mutations outside of the N-terminal region also affect PB2-MAVS binding [85–87]. One of those mutations is PB2 T588I, which was identified in a swine isolate of the 2009 pandemic H1N1 virus strain (pdm09) that was highly pathogenic in mice [85]. Upon closer examination, the T588I mutation was found to improve polymerase activity and increase viral replication in cell culture and murine lungs. Interestingly, the mutation also led to a decrease in IFN-β expression [85]. This decrease correlated with the stronger binding of the T588I mutant to MAVS [85]. Another mutation implicated in MAVS binding, and gaining prevalence among circulating avian H9N2 viruses in recent years, is I292V in the Mid-link domain of PB2 [86]. I292V improved PB2-MAVS binding and decreased IFN-β expression, resulting in a more severe disease in mice [86]. Although it is unknown how PB2 inhibits innate immune activation upon MAVS binding, it is possible that PB2 prevents a correct subcellular localisation of MAVS, limits MAVS oligomerization, or induces MAVS degradation [88, 89].

Another mechanism through which PB2 modulates MAVS-mediated signalling is by targeting TNF receptor-associated factor 3 (TRAF3), the adaptor protein of MAVS that is required for optimal signal transduction [90]. TRAF3 interacts with MAVS (Fig. 2), catalysing recruitment of the TBK1 and IKKε kinases, which in turn phosphorylate IRF3 promoting expression of IFN genes [90, 91]. To achieve this, TRAF3 needs to be activated by TRIM35 through the addition of Lys63-linked polyubiquitin. PB2 prevents polyubiquitination of TRAF3 by binding to TRAF3 with PB2 residues 490–759. The binding between PB2 and TRAF3 disrupts formation of the TRAF3-MAVS complex, and inhibits downstream IFN-β promoter activation. TRIM35, on the other hand, counters this immunomodulatory activity of PB2 by adding Lys48-linked polyubiquitin to Lys736 of PB2, which targets PB2 for proteasomal degradation [90].

The PA subunit of the viral RNA polymerase also inhibits innate immune signalling by binding to IRF3. This interaction prevents phosphorylation and nuclear translocation of IRF3, both of which are central for IFN expression [92]. The same study also showed that a D108A mutation in PA inhibits PA-IRF3 interaction. However, it is unclear how D108 could be involved in this interaction as it is a catalytic residue of the endonuclease and not located on the surface of the domain.

### PB2 is targeted to mitochondria

Mitochondria serve as platform for the interaction between RIG-I and MAVS and thus play a prominent role in innate immune signalling [88, 93, 94]. Influenza A viruses encode an accessory protein, called PB1-F2, that specifically localises to mitochondria and interferes with their function in innate immune signalling [26]. However, there is also a small pool of PB2 that localises to the mitochondrial matrix. The purpose of this localisation remains controversial [84, 95–97].

The mitochondrial targeting signal of PB2 has been mapped to PB2 residues L7 and L10, or N9 [84, 95]. It has also been shown that the PB2 proteins of different influenza A virus strains have different mitochondrial localisation tendencies, with seasonal human strains localising to mitochondria and avian strains showing reduced localisation, suggesting that mitochondrial localisation might play a role in host adaptation [84]. The strain-specific localisation of PB2 dependents on the PB2 residue nine, which is typically an aspartate (D) in the PB2 proteins of avian strains and an asparagine (N) in the PB2 proteins of human-adapted strains. In vitro, introduction of an avian-like N9D mutation in the PB2 of the A/WSN/33 virus strain increases IFN-β expression in lung epithelial cells. In mice, however, the N9D mutation decreased IFN-β expression, likely because of the impaired growth of the N9D mutant in vivo [84]. Presently, the mechanisms behind innate immune modulation by the mitochondrial PB2 remain unclear, but they might involve changes in mitochondrial dynamics or mitochondrial membrane potential [95, 96].

## Aberrant replication products as innate immune activators

### Aberrant replication products and their synthesis

**Fig. 4:**
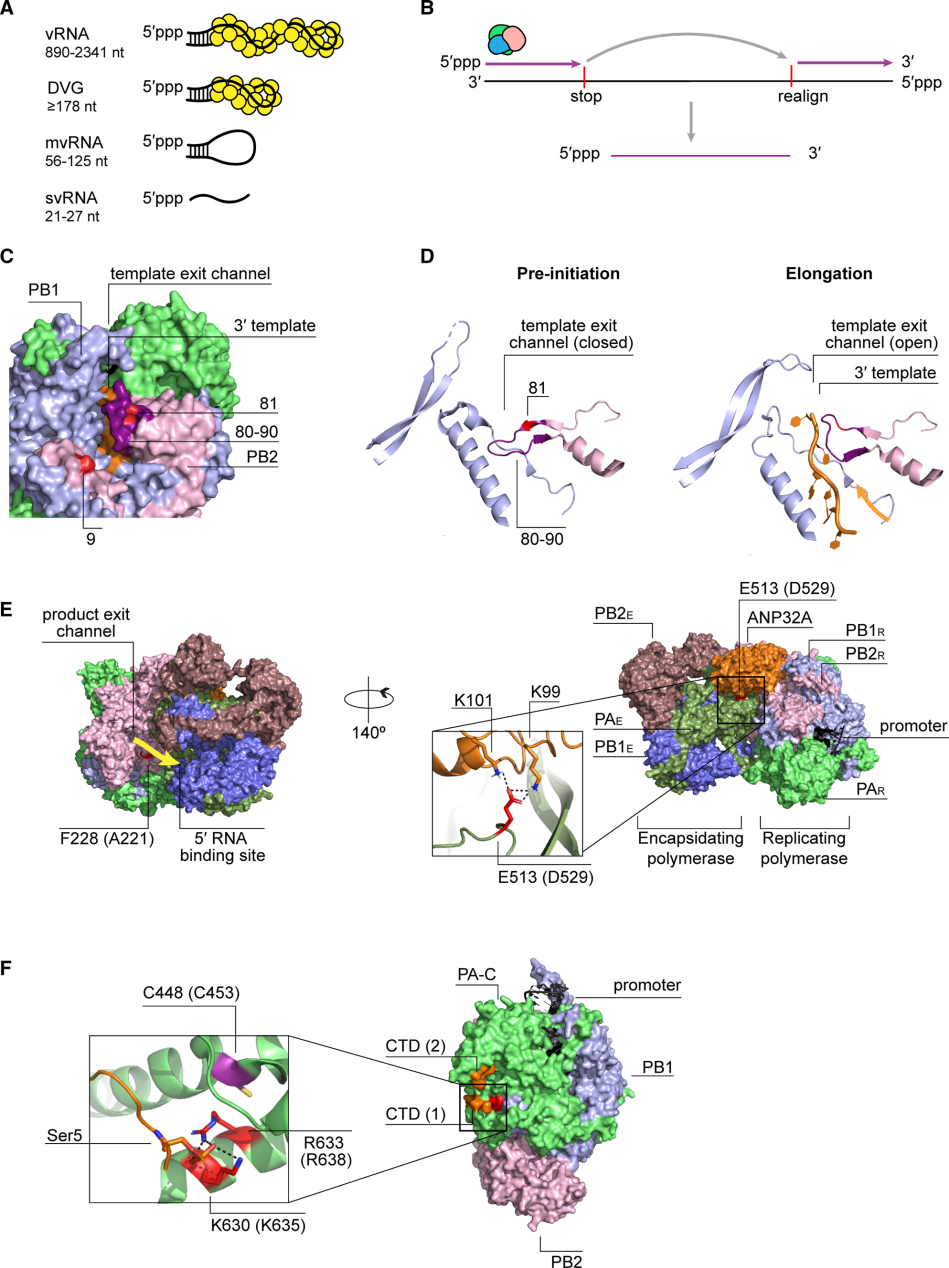
Aberrant viral RNA formation and RNA polymerase residues putatively implicated in this process. **A** A schematic representation of different types of aberrant RNAs produced by the influenza A virus polymerase. **B** A possible mechanism for the DVG and mvRNA synthesis during replication. **C** A surface representation of the bat H17N10 influenza A polymerase (PDB 6T0V) during transcription elongation. The 3′ terminus of the template (orange) can be seen exiting through the template exit channel. PB2 residues 9 and 81 are highlighted in red and PB2 80–90 loop is shown in purple. **D** A cartoon representation of the template exit channel of the bat H17N10 influenza A polymerase at the stage of transcription pre-initiation (PDB 6T0N) and during transcription elongation (PDB 6T0V). The 80–90 loop (purple) undergoes an outward movement, allowing opening of the template exit channel during elongation. Direction of the exiting template is indicated with an arrow. **E** A surface representation of the replicating-encapsidating polymerase dimer of the influenza C virus (ICV) polymerase in the complex with chicken ANP32A (PDB 6XZR). Residue numbering is as in ICV RNA polymerase. The corresponding IAV RNA polymerase residues are shown in parenthesis. PB2 residue F228 of ICV (A221 IAV) is located in an RNA path (yellow arrow) between the product exit channel of the replicating polymerase and the 5′ binding site of the encapsidated polymerase. Hydrogen bonds between the PA E513 of ICV (D529 in IAV) and two lysines of ANP32A (K99 and K101) are shown. **F** A surface representation of the bat H17N10 influenza A virus bound to the CTD of Pol II (PDB 5M3H). Two binding sites for Pol II CTD (orange) are shown. Residue numbering as in bat IAV, and corresponding human and avian residues are shown in parenthesis. PA residues, K630 and R633 of the bat IAV polymerase (corresponding to K635 and R638 of the human or avian IAV polymerase, respectively) are shown to form hydrogen bonds with phosphorylated Ser5 of the Pol II CTD. PA residue C448 in the bat IAV polymerase (C453 in the human or avian IAV) is indicated in purple

Besides full-length replication products, the influenza virus RNA polymerase also generates aberrant replication products, including DVGs, mvRNAs, and svRNAs (Fig. 4A) [24, 98–100]. svRNA are 21–27 nucleotides long and only contain the 5′ terminus of the vRNA template. By contrast, DVGs and mvRNAs both contain the conserved 5′ and 3′ terminal ends that are present in each vRNA segment and bound by the viral RNA polymerase. However, they lack internal sequences and can be distinguished from full-length vRNAs by their size, with DVGs being typically 178 to several hundred nucleotides long, and mvRNAs being 56–125 nucleotides long [24, 101, 102].

vRNAs, DVGs and mvRNAs all contain the conserved RNA promoter structure with 5′ triphosphate that forms a ‘panhandle’ in solution [103]. In vitro and in vivo, this RNA structure is recognised by the cellular RIG-I sensor and able to activate the MAVS signalling cascade [7, 19, 24, 66, 104]. RIG-I binds different influenza A virus RNA species with different efficiencies. Aberrant RNAs of 56–125 nucleotides long are bound by RIG-I more efficiently than longer aberrant RNAs, and shorter vRNA segments are bound more efficiently than longer vRNA segments [24, 104]. Interestingly, aberrant RNAs shorter than 56 nucleotides are not bound by RIG-I at all, even though short artificial hairpins are potent RIG-I agonists [24, 105]. Although, both mvRNAs and DVGs are potent inducers of IFN expression, they are thought to have opposite effects on disease, with mvRNAs having been linked to virulence and the cytokine storm, both common for the highly pathogenic influenza virus strains, and DVGs to protective IFN responses and a reduction of viral virulence [24, 106, 107].

The molecular mechanisms underlying DVG and mvRNA formation are currently poorly defined. In one model, the internal deletions are generated when the viral RNA polymerase pauses during elongation, back-tracks to separate template and nascent strand, and finally translocates to a downstream template sequence to realign the nascent strand and continue nascent RNA extension (Fig. 4B) [22, 98]. Such polymerase translocations might be affected or directed by A/U-rich sequences, which have been observed near DVG breakpoints [108, 109]. It is possible that such A/U-rich sequences facilitate separation of the template and nascent strand prior to translocation. Other models for DVG and mvRNA synthesis, which involve, for instance, endonucleolytic cleavage and ligation of the product RNA, are not supported by experimental data [98].

Several viral factors have been associated with the formation of aberrant RNAs. Recent studies have shown that elongation defects can be induced experimentally by limiting the availability of NP, an important elongation factor and key component of RNPs, suggesting that impaired elongation or aberrant encapsidation play a role in DVG or mvRNA formation [24, 110, 111]. In addition, mutations in several viral proteins, such as nuclear export protein, matrix protein 1 and 2, and the RNA polymerase subunits, also promote the formation of DVGs [34, 112–114].

### RNA polymerase mutations that affect aberrant RNA synthesis

We can learn more about the potential mechanisms underlying aberrant RNA generation and their role in innate immune activation by studying the RNA polymerase mutations that affect their formation. High levels of mvRNA production by the polymerases of highly pathogenic avian H5N1 and 1918 pandemic viruses are in part determined by avian-adaptive mutations in the PB2 polymerase subunit of those strains [24]. Introducing such avian-specific PB2 mutations, e.g., N9D and M81T, into the lab-adapted A/WSN/33 (H1N1) strain significantly increased mvRNA production and IFN-β promoter activation [24]. Both these residues are located at the top of the RNA polymerase core, near the interaction interface of the PB1 C-terminus and the PB2 N-terminus (Fig. 4C). Of the two residues, PB2 residue 81 stands out as it is located within the PB2 80–90 loop that undergoes an outward conformational change to allow template egress during elongation (Fig. 4D) [41]. Because of the role of the 80–90 loop in elongation, it is tempting to speculate that a mutation of residue 81 could trigger elongation defects, which may contribute to mvRNA production.

Synthesis of aberrant RNAs is also affected by the fidelity of the viral polymerase. A V43I mutation in the PB1 subunit, which reduces the mutation rate of RNA synthesis by approximately twofold in some genetic backgrounds, was also shown to lower levels of mvRNA synthesis by the polymerases of the 1918 pandemic and H5N1 strains [24, 115]. Interestingly, V43I change in H5N1 strain also decreased neurotropism and lowered lethality in mice [116]. V43I is located near the NTP entry channel of the polymerase and may increase polymerase fidelity by improving nucleoside selectivity [116]. However, it remains unclear whether the same mechanisms could also contribute to the production of aberrant RNA species.

Similar to mvRNA synthesis, DVG formation is affected by mutations in the RNA polymerase. Two of these mutations, PB2 A221T and PA D529N, were identified in a virus isolated from a fatal case of pdm09 (H1N1) influenza [114]. Interestingly, the two mutations demonstrated opposite effects on DVG generation and immune activation when studied in more detail. PB2 A221T increased DVG formation and enhanced protective antiviral responses, while PA D529N counteracted both effects [114]. Analysis of the localisation of PA A221 in various influenza virus RNA polymerase structures shows that this residue can be involved in the interaction between the N-terminal and the 627 domains of PB2 (PDB: 6T0V) or it can be residing in the path that the nascent RNA takes when it emerges from the replicating polymerase to bind the encapsidating polymerase (PDB: 6XZR) (Fig. 4E). While it is not clear if those localisations are directly involved in DVG production, it is tempting to speculate that the A221T mutation could affect encapsidation of the nascent RNA, thereby reducing processivity. Alternatively, the mutation may lead to aberrant RNA formation by affecting NP recruitment to the nascent strand, which was proposed to occur near the RNA transition path [48]. By contrast, PA D529 resides above the Pol II binding interface of the transcriptionally active polymerase (PDB: 6T0V), while in the ANP32A-supported dimer, PA D529, is located at the ANP32A interaction interface (Fig. 4E) [48]. Thus, in the dimer, PA D529N is ideally positioned to compensate for defects in polymerase processivity or encapsidation by stabilizing ANP32A binding and RNA polymerase dimer formation.

Aberrant polymerase activity can also be the result of defects in viral transcription. Influenza virus transcription is dependent on cap-snatching and the binding of the C-terminal domain of PA to a Ser5 phosphorylated CTD of Pol II. Mutations in key PA residues involved in this interaction, K635A and R638A (Fig. 4F), not only decrease the activity of the A/WSN/33 (H1N1) polymerase, but also promote DVG formation [108, 113, 117]. On the other hand, a PA C453R mutation at same site (Fig. 4F) reduces DVG formation, because it may restore the binding of PA to the Pol II CTD, as suggested by the structural analysis [113, 117]. What might be the mechanism behind DVG formation in this case? As mentioned above, elongation defects can be induced experimentally by limiting the availability of NP during viral replication [24, 110, 111]. Although not experimentally confirmed, the transcriptional defects induced by reduced Pol II binding likely result in lower NP levels, which subsequently promote aberrant polymerase activity [24, 110, 111].

### Host-adaptive polymerase mutations improve polymerase activity and increase innate immune activation

The influenza A virus RNA polymerase plays a major role in the adaptation of avian influenza virus strains to mammalian cells. Several major host-adaptive mutations induce a strong activation of innate immune responses which correlates with the improvement in viral replication.

**Fig. 5:**
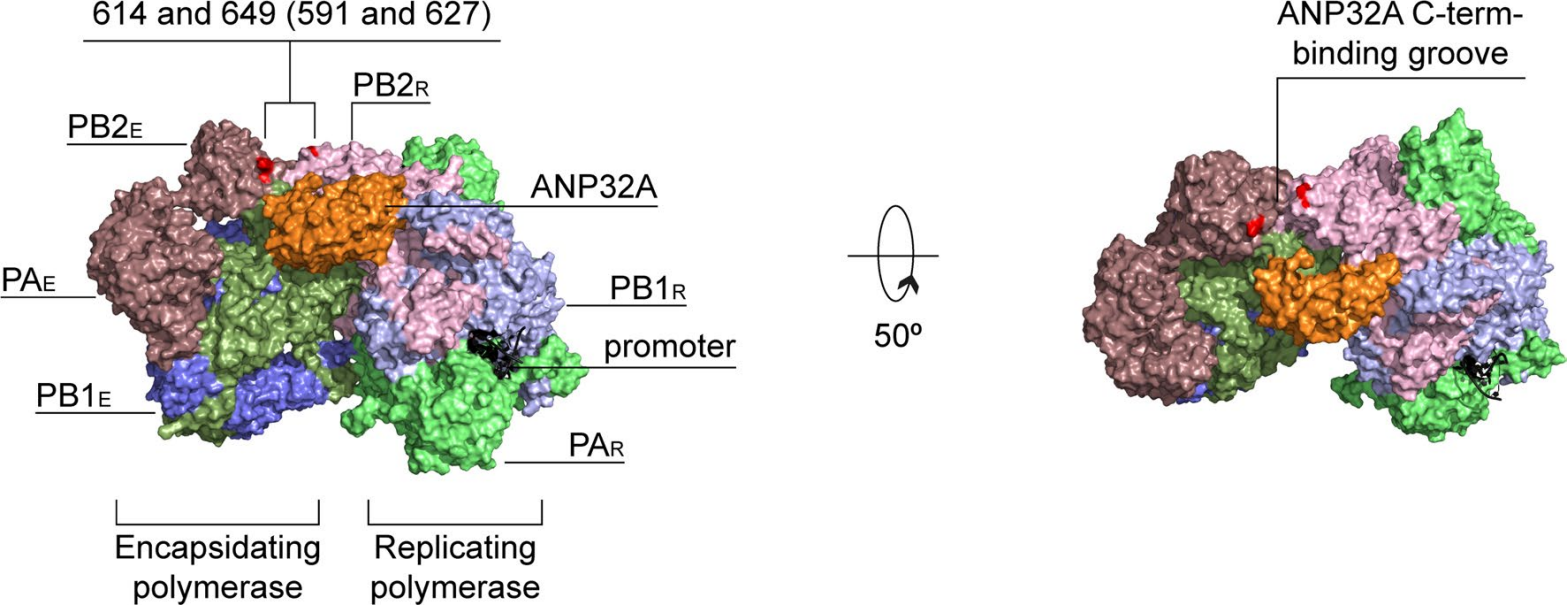
Host-adaptive immunostimulatory mutations. A surface representation of the replicating-encapsidating polymerase dimer of the ICV polymerase in the complex with chicken ANP32A (PDB 6XZR). PB2 residues 614 and 649 of ICV are highlighted in red and correspond to residues 591 and 627 of IAV shown in parenthesis

One of the most well-studied polymerase adaptations is a E-to-K mutation at residue 627 of PB2 [118]. This mutation is located in the PB2 627-domain (named after the 627 mutation; Fig. 5) and able to improve the activity of avian-adapted influenza A virus polymerases in mammalian cells [31]. This improvement results in higher viral loads, extra-respiratory spread and enhanced virulence of the E627K-containing avian viral strains in mice [119–123]. In the majority of cases, the increased virulence of the 627K-containing avian viruses is also accompanied by an overproduction of pro-inflammatory cytokines, persistent neutrophil infiltration and delayed lymphocyte recruitment, which are all hallmarks of the cytokine storm [119–124].

Recent experiments have shown that a lysine at position 627 is essential for a stable interaction of the viral RNA polymerase with mammalian host-cell protein ANP32A [50]. ANP32A had previously been proposed to be essential for the synthesis of vRNA from a cRNA template [53], but recent structural and biochemical evidence suggests that it brings together the replicating and encapsidating polymerases in a viral replicase complex that can synthesise both vRNA or cRNA [48, 49]. The ANP32A-supported dimer shows that the basic K627, but not the acidic E627, can efficiently interact with the C-terminal low-complexity acidic region of mammalian ANP32A (Fig. 5) [48]. By contrast, avian ANP32A homologs contain an additional exon, and can interact with the polymerase dimer when the polymerases in the dimer contain an E627 [50, 125].

In some avian influenza virus strains, a Q591K mutation in the PB2 627 domain [G590S and Q591R in pdm09 (H1N1)] can compensate for the absence of the E627K signature and support the activity of avian-adapted polymerases in mammalian cells [126, 127]. Like PB2 residue 627, residue 591 is located at the binding site for the C-terminal region of ANP32A (Fig. 5) and introduction of a basic amino acid at this site is thought to improve the interaction between the polymerase and mammalian ANP32A [48]. By stabilising the replicase dimer, the Q591K mutation increases the replication of the avian H7N9 and H9N2 viruses in murine lungs, inducing strong inflammatory responses and enhancing virulence as a result [122, 123, 128].

PB2 mutation D701N has also been associated with improved replication and higher levels of innate immune activation [31, 123, 128]. However, unlike the mutations discussed above, amino acid 701 resides in the PB2 NLS and promotes replication by improving nuclear import of PB2 and vRNPs [129–131].

### An emerging role of the Mid-link domain of PB2 in innate immune activation

**Fig. 6:**
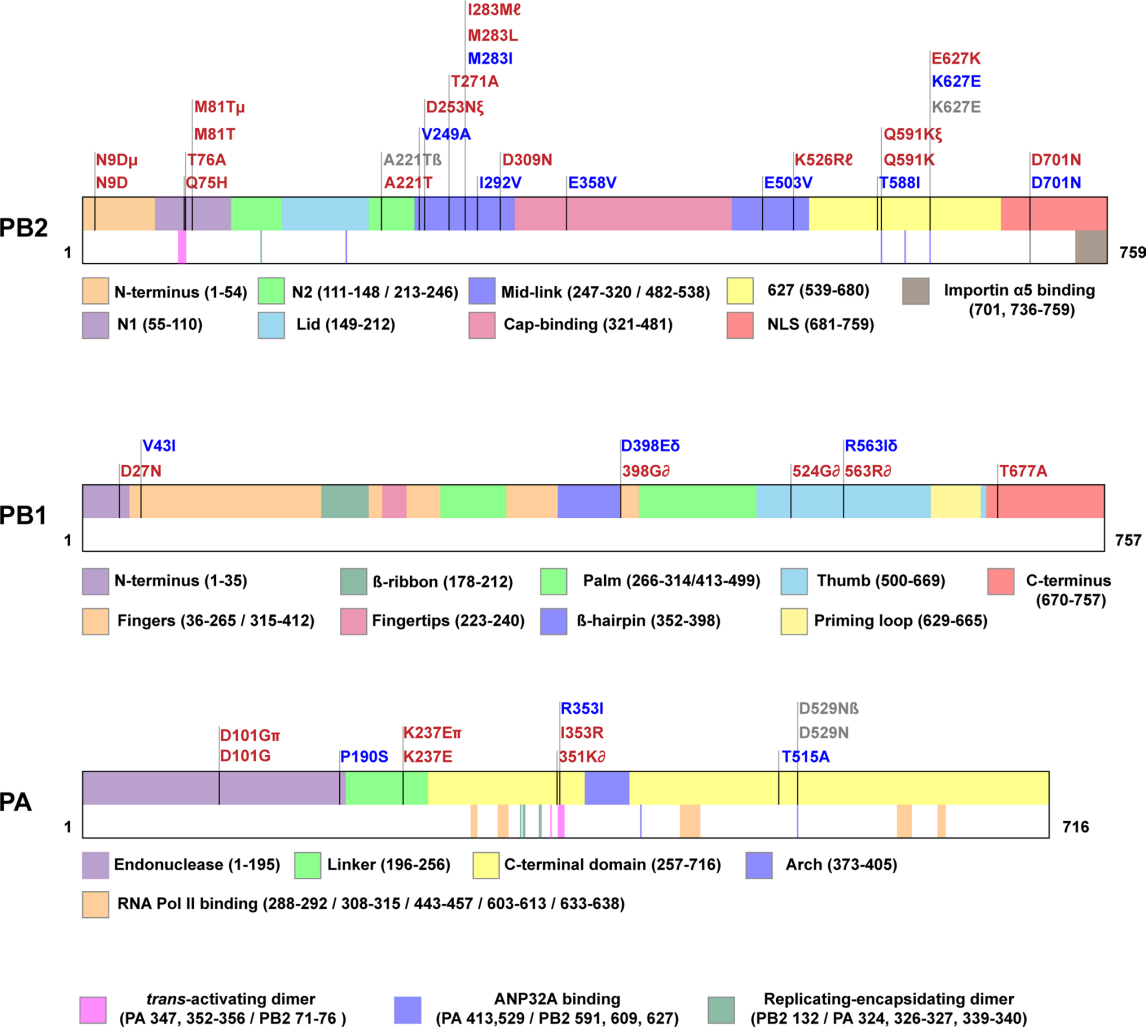
Immunostimulatory and immunoinhibitory mutations in the influenza A virus RNA polymerase. Polymerase mutations that affect innate immune activation (Supplementary Table 2) were mapped to the polymerase subunits of the influenza A virus. Mutations that induced higher innate immune activation in comparison to the background strain are shown in red and those that decreased innate immune activation are shown in blue. Mutations that did not affect innate immune responses in comparison to the background virus strain are shown in grey. Combinations of mutations are marked with Greek symbols. The structural domains in each subunit are indicated in different colours. Additionally, functional regions involved in importin binding [131], RNA Pol II interactions [117] (RNA Pol II binding sites differ for influenza B and C viruses [44]), formation of polymerase dimers [48, 56] and ANP32A-binding [48] are highlighted in the bottom half of each segment

To explore the distribution of published polymerase mutations that affect innate immune responses and identify potentially novel clusters of interest, we mapped those mutations to the polymerase subunits (Fig. 6, Supplementary Table 2). The PB2 subunit contained the majority of the identified immunostimulatory or immunoinhibitory mutations (Fig. 6). In particular, a number of these mutations cluster in the Mid-link domain of PB2 (residues 247–320/482–538). This clustering indicates that Mid-link might play an important role in polymerase’s function and innate immune recognition of the viral infection, yet this region has presently not been assigned a specific role in viral genome replication or transcription. What could the role of the Mid-link domain and its mutations be?

**Fig. 7:**
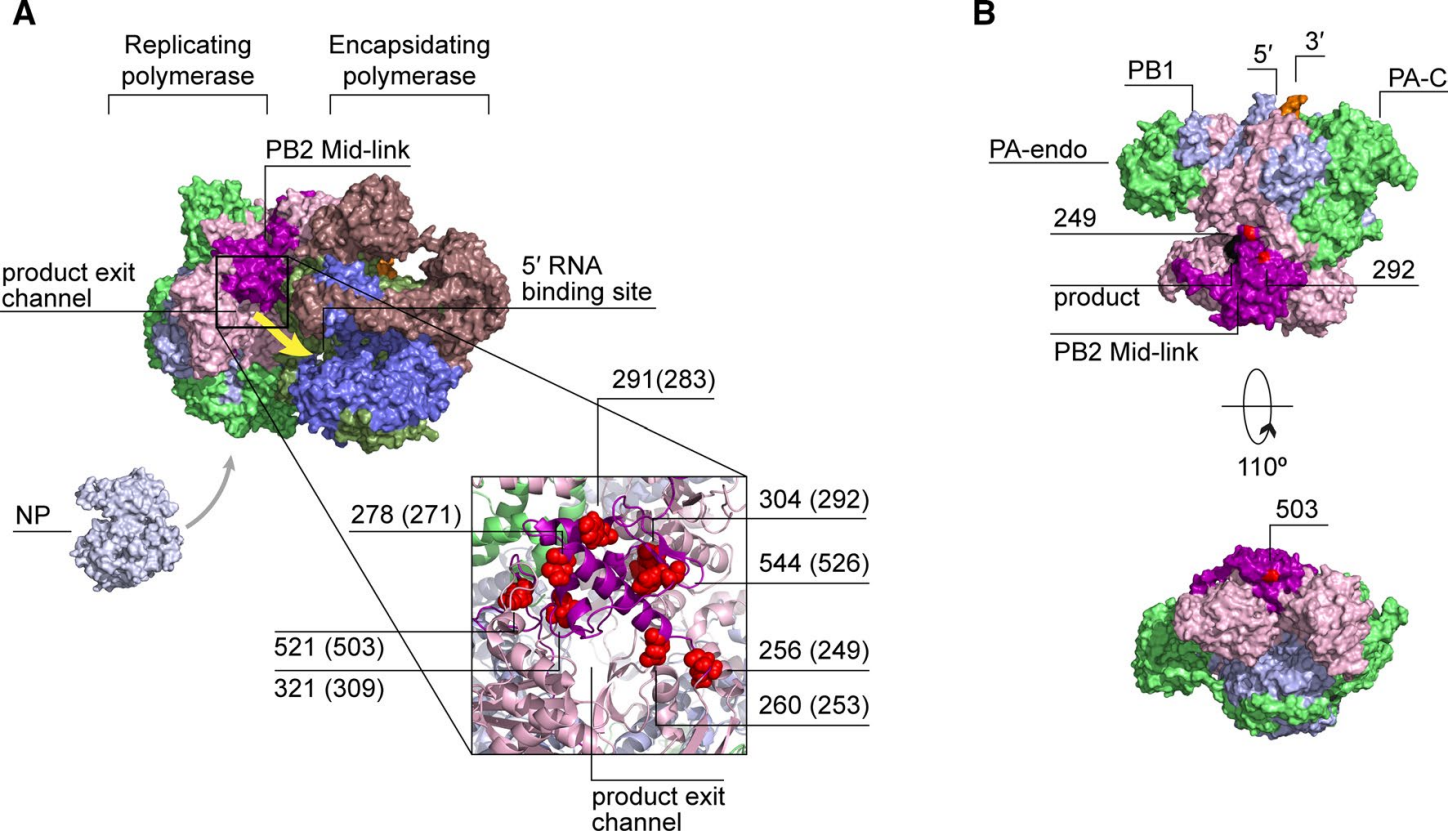
Mid-link domain mutations. **A** A surface representation of the encapsidating-replicating polymerase dimer of ICV (PDB 6XZR). The Mid-link domain of PB2 is shown in violet. The movement of the 5′ end of the nascent RNA from the product exit channel of the replicating polymerase to the 5′ binding site of the encapsidating polymerase is shown with a yellow arrow. Mid-link residues, whose alteration affects innate immune activation, are shown as red spheres (residue numbering is as in ICV, corresponding IAV amino acids are shown in parenthesis). **B** Localisation of the IAV Mid-link residues 249, 292 and 503 (shown in red) on the structure of the human H3N2 influenza A virus RNA polymerase (PDB 6RR7)

The PB2 Mid-link domain might play a key role in stabilizing the conformational rearrangements of the RNA polymerase as it transitions from one state to another or in encapsidation of the nascent RNA strand. It forms extensive, transient interactions with the flexible domains of PA and PB2 in various conformations of the viral polymerase. In addition, analysis of the ANP32A-supported replicase suggests that the Mid-link domain may play a role in nascent strand egress or encapsidation. In the dimer, residues 252–273 and 519–523 (influenza C virus PB2 residues 259–280 and 538–542, respectively) of the replicating polymerase face the encapsidating polymerase but are not directly interacting with it. Instead, these residues face a groove that separates the two polymerases in the dimer and which the emerging 5′ terminus of the nascent strand must bridge to reach the promoter binding pocket of the encapsidating polymerase (Fig. 7A). It is tempting to speculate that these residues of the Mid-link domain play a role in NP recruitment or the encapsidation of the nascent strand [48].

Mid-link domain might also play a role in adaptation as several mutations in the Mid-link domain were shown to improve polymerase activity and the replication of avian influenza virus strains in mammalian cells. For instance, the well-documented adaptive mutation Q591K in the 627 domain occurs together with the Mid-link mutation D253N in an avian H9N2 virus [132]. In this strain, D253N improves polymerase activity, enhances viral replication in mice, and stimulates interferon and pro-inflammatory cytokine production [132]. Similarly, Mid-link mutation T271A in the RNA polymerase of an avian H7N9 isolate partially compensates for the lack of a PB2 E627K mutation in mammalian cells, improving polymerase activity and viral replication, while at the same time increasing pro-inflammatory cytokine expression [128]. Moreover, in H5N1 isolates, a M283L mutation was shown to increase polymerase activity, viral replication and innate immune activation in mice, whereas a M283I mutation decreased these properties [133]. Similarly, the reverse I283M mutation in the combination with K526R in the avian H5N8 virus significantly upregulated innate immune activation in murine lungs [134]. Thus, mutations in the Mid-link domain might induce innate immune activation by improving polymerase activity, similar to the other adaptive mutations.

The Mid-link domain could also have a stand-alone immunomodulatory function. Several mutations in this region supressed innate immune activation despite improving polymerase activity. Two of them, V249A and I503V, arose in a recombinant A/PR/8/34 (H1N1) virus, which contained a dysfunctional NS1 of the bat influenza A virus [135]. The mutations were able to compensate for the absence of innate immune modulation by NS1, reducing IFN induction, despite simultaneously increasing viral replication [135]. The I292V mutation in the avian H9N2 virus, which increases binding to MAVS, also suppressed innate immune activation despite higher activity of the mutant polymerase [86]. All three mutations reside in the solvent-exposed region of the Mid-link domain (Fig. 7B) and may thus be able to inhibit innate immune activation by interacting with the components of the host innate immune system.

## Conclusions and outstanding questions

The influenza A virus RNA polymerase plays multiple roles in the innate immune response to influenza virus infection. Not only can the subunits of the RNA polymerase affect innate immune activation by interfering with the components of the cellular signalling pathways, the enzyme can also produce immunostimulatory RNA species. These activities of the RNA polymerase are not unique to influenza A viruses, as RNA polymerases of viruses belonging to *Flaviviridae*, *Picornaviridae* and *Coronaviridae* families are also known to specifically target and inhibit innate immune signalling [136–139], while production of aberrant RNA species has been described for the majority of RNA viruses as well [140].

Despite recent advances in our understanding of the structure and the immunostimulatory and immunoinhibitory activities of the influenza A virus polymerase, many fundamental questions about the molecular mechanisms involved remain unanswered. These include, but are not limited to, (i) how does RIG-I gain access to viral RNA (and the viral RNA termini in particular) in the context of a fully assembled RNP; (ii) what is the molecular mechanism underlying the generation of DVGs and mvRNAs; (iii) why do aberrant viral RNAs trigger innate immune responses more efficiently than full-length viral RNA segments; (iv) does aberrant RNA synthesis confer any evolutionary advantage, and (v) are the immunosuppressive and enzymatic activities of the viral polymerase separated in space and time? To find answers to those questions, existing and novel RNA polymerase mutants can be used. Screening approaches in combination with next generation sequencing have proven to be particularly powerful for the identification of such immunostimulatory RNA polymerase mutants [33, 141]. However, biochemical and molecular research is still needed to better understand how such mutations affect polymerase function.

The immunomodulatory or -stimulatory activity of the viral polymerase could also guide the development of novel antiviral treatments. Even though current drugs targeting the influenza virus polymerase primarily focus on blocking its activity, targeting its immunomodulatory function could potentially have an added benefit of activating the host’s natural immune defence during infection. However, care must be taken to not over-stimulate the innate immune response. Additionally, DVG-containing influenza viruses or cloned DVGs have been proposed as both influenza-specific and a broad-spectrum antiviral treatment due to their interfering and immunostimulatory activity [106, 142].

The knowledge of the processes by which influenza virus polymerase stimulates or inhibits innate immune responses can also be used in rational vaccine design. Several PB1, PB2 and NP mutations in the current live-attenuated influenza vaccine confer its cold-adapted, attenuated and temperature-sensitive phenotype [143], while alterations in NS1 protein have been explored as a novel approach to improve efficacy of the live-attenuated vaccines [144, 145]. Recent studies also showed that addition of immunostimulatory polymerase mutations, for instance in combination with mutations or deletions in NS1, improves vaccine immunogenicity and protection against infection [141, 146]. DVG-containing (interfering) vaccines were also shown to protect mice and ferrets from severe influenza infection [147, 148]. However, the presence of DVGs in the live-attenuated vaccines has been suggested to reduce their immunogenicity by interfering with the replication of the vaccine strain [149, 150], suggesting that a careful balance may need to be found for some of the above approaches. Understanding the viral and host molecular determinants of DVG production can therefore help to regulate aberrant polymerase activity of the vaccine strains. In this way, the multifunctional role of the viral RNA polymerase in innate immune activation represents an important area of future research.

## Supplementary Information

Below is the link to the electronic supplementary material.

Supplementary file 1 (DOCX 210 KB)

## Data Availability

Not applicable.
