# Supplementary material for: The influenza virus RNA polymerase as an innate immune agonist and antagonist
Source: Cell Mol Life Sci. 2021 Oct 22;78(23):7237–56. doi: 10.1007/s00018-021-03957-w (PMC8532088; doi:10.1007/s00018-021-03957-w)
Supplement: Supplementary file 1 — (DOCX 210 KB) [file 18_2021_3957_MOESM1_ESM.docx]

**Supplementary Table 1.** Reassortments of the Influenza A virus polymerase segments and their effect on innate immune responses, polymerase activity, viral growth and virulence.

| Publication | Segment | Segment origin | Background virus | Immune response in comparison to background virus | Polymerase activity in comparison to background virus | Viral growth in comparison to background virus | Virulence in mice in comparison to background virus |
| --- | --- | --- | --- | --- | --- | --- | --- |
| Mok et al. [1] | PB2 | A/WSN/33 (H1N1) | A/Vietnam/1203/04 (H5N1) | Decrease in TNF-α, IP-10 and IFN-β expression in primary human macrophages | No change in M mRNA levels in primary human macrophages | No change in M mRNA levels in primary human macrophages | N/A |
| Liniger et al. [2] | PB2 | A/chicken/Yamaguchi/7/04 (H5N1) HPAIV | A/duck/Hokkaido/Vac-1/04 (H5N1) LPAIV (with HA of A/chicken/Yamaguchi/7/04 (H5N1)) | Decrease in type I IFN production in HD-11 cells | N/A | No change in viral titres in HD-11 cells | N/A |
| Watanabe et al. [3] | PB2 | A/whistling swan/Shimane/580/2002 (H5N3) LPAIV | A/ck/Yamaguchi/7/2004 (H5N1) HPAIV | Increased expression of CCl20 and IL-8 in LMH cells | N/A | No change in growth in LMH cells | N/A |
| Liniger et al. [2] | PB2 | A/duck/Hokkaido/Vac-1/04 (H5N1) LPAIV | A/chicken/Yamaguchi/7/04 (H5N1) HPAIV | Increase in type I IFN production in HD-11 cells | N/A | No change in viral titres in HD-11 cells | N/A |
| Forero et al. [4] | PB2 | A/Brevig Mission/1/18 (H1N1) | 1918-like avian virus (Watanabe et al. [5]) | Increased inflammatory responses and recruitment and activation of inflammatory monocytes, macrophages and activated NK cells in mice. Inhibition of Wnt signalling involved in lung repair pathways | N/A | Increased viral growth in murine lungs | Increased virulence (decreased MLD_50_) |
| Williams et al. [6] | PB2 | A/California/04/2009 (H1N1) | A/shorebird/Delaware/22/2006 (H7N3) | Increased levels of inflammatory cytokine production, such as IL-10, CCL2, CL3, CCL5, and TNF-α | Increased activity | Increased viral growth in mice at 3 days post-infection, but not 7 days post-infection | Increased virulence (weight loss) |
| Park et al. [7] | PB2 | A/EM/Korea/W149/06 (H5N1) HPIAV | A/MD/Korea/W452/2014 (H5N8) LPIAV | Increased expression of proinflammatory cytokines, such as TNF-α, lL-1β, IL- 6, IL-18, and GM-CSF at 3 and 5 days post-infection in mice | Increased activity | Increased viral growth in MDCK, A549 and NHBE cells | Increased virulence (weight loss) and mortality |
| Mok et al. [1] | PB2 | A/Vietnam/1203/04 (H5N1) | A/WSN/33 (H1N1) | No change in TNF-α, IP-10 and IFN-β expression in primary human macrophages | No change in M mRNA levels in primary human macrophages | No change in growth in primary human macrophages | N/A |
| Liniger et al. [2] | PB1 | A/chicken/Yamaguchi/7/04 (H5N1) HPAIV | A/duck/Hokkaido/Vac-1/04 (H5N1) LPAIV (with HA of A/chicken/Yamaguchi/7/04 (H5N1)) | Decrease in type I IFN production in HD-11 cells | N/A | No change in viral titres in HD-11 cells | N/A |
| Li et al. [8] | PB1 | A/Indonesia/5/05 (H5N1) | A/WSN/33 (H1N1) | Increase in TNF-α, IFN-β, RANTES and IP-10 gene expression in primary human macrophages and IP-10 and RANTES in primary human pneumocytes | Increased activity in a luciferase reporter assay | Increase in growth only at 8 h post-infection in MDCK, no difference at later time points | N/A |
| Watanabe et al. [3] | PB1 | A/whistling swan/Shimane/580/2002 (H5N3) LPAIV | A/ck/Yamaguchi/7/2004 (H5N1) HPAIV | Increased expression IL-8 in LMH cells, no change in CCL20 expression | N/A | No change in growth in LMH cells | N/A |
| Liniger et al. [2] | PB1 | A/duck/Hokkaido/Vac-1/04 (H5N1) LPAIV | A/chicken/Yamaguchi/7/04 (H5N1) HPAIV | No change in type I IFN production in HD-11 cells | N/A | No change in viral titres in HD-11 cells | N/A |
| Hu et al. [9] | PA | A/Goose/Jiangsu/k0403/2010 LPAIV | A/Chicken/Jiangsu/k0402/2010 HPAIV | Decreased expression of IL-6, Mx1, CXCL10 and CXCL11 in murine lungs | Decreased activity in 293T cells | Decreased titres in murine spleen, brain and lungs | Decreased virulence (increased MLD_50_) |
| Wang et al. [10] | PA | A/Cambodia/P0322095/2005 (H5N1) | A/WSN/33 (H1N1) | Increase in IFN-β expression in 293T cells and earlier apoptosis in MDCK cells | Increased activity *in vitro* and in a replicon assay | Increase in growth in a single-cycle infection in MDCK and Vero cells; decrease in multicycle infection in cells and during infection in mice. | Decreased virulence (lower mortality) |
| Sakabe et al. [11] | PA^a^ | A/Vietnam/ UT3028II/03 clone 2 (H5N1) | A/Indonesia/UT3006/05  (H5N1) | Increase in cytokine expression, such as IL-6, IP-10, MIP-1α, MIP-1β, RANTES, TNF-α, and MIG in human macrophages | No change in polymerase activity in reporter assay | No change in viral growth in human macrophages | Increased virulence (higher mortality) |
| Hu et al. [9] | PA | A/Chicken/Jiangsu/k0402/2010 HPAIV | A/Goose/Jiangsu/k0403/2010 LPAIV | Increased expression of IL-6, Mx1, CXCL10 and CXCL11 in murine lungs | Increased activity in 293T cells | Increased growth in murine brain and lungs | Increased virulence (decreased MLD_50_) |
| Dlugolenski et al. [12] | PA^a^ | A/swine/Illinois/02860/09 (swH1N2) | A/California/04/09 (huH1N1) | Increased levels of chemoattractant MIP-2 and increased infiltration of neutrophils and NK cells in the murine lungs | N/A | No change in viral growth in mice | N/A |
| Watanabe et al. [3] | PA | A/whistling swan/Shimane/580/2002 (H5N3) LPAIV | A/ck/Yamaguchi/7/2004 (H5N1) HPAIV | No change in IL-8 and CCL20 expression in LMH cells | N/A | No change in growth in LMH cells | N/A |
| Vigeveno et al. [13] | PB2 and PA | A/Eurasian Wigeon/Netherlands/4/2016 (H5N8) HPIAV | A/Chicken/Netherlands/emc-3/2014 (H5N8) LPIAV (PB1 and NP only) | Decreased IFN-β reporter activity in HEK293T cells | Decreased activity in HEK293T and QT6 cells | N/A | N/A |
| Vigeveno et al. [13] | PB2 and PA | A/Chicken/Netherlands/emc-3/2014 (H5N8) LPIAV | A/Eurasian Wigeon/Netherlands/4/2016 (H5N8) HPIAV (PB1 and NP only) | Increased IFN-β reporter activity in HEK293T cells | Increased activity in HEK293T and QT6 cells | N/A | N/A |
| Liedmann et al. [14] | PB1 and PA | A/Puerto Rico/8/34 (H1N1) variant M | A/Puerto Rico/8/34 (H1N1) | Increased phosphorylation of STAT1 in A549 cells | N/A | N/A | N/A |
| Liniger et al. [2] | 3P | A/chicken/Yamaguchi/7/04 (H5N1) HPAIV | A/duck/Hokkaido/Vac-1/04 (H5N1) LPAIV (with HA of A/chicken/Yamaguchi/7/04 (H5N1)) | Decrease in type I IFN production in HD-11 cells | N/A | No change in viral titres in HD-11 cells | N/A |
| Vigeveno et al. [13] | 3P | A/Eurasian Wigeon/Netherlands/4/2016 (H5N8) HPIAV | A/Chicken/Netherlands/emc-3/2014 (H5N8) LPIAV | Decrease in IFN-β expression in DEF and DLH cells | Decreased activity in HEK293T and QT6 cells | Decrease in viral titres in DEF cells | N/A |
| Mok et al. [1] | 3P | A/Vietnam/1203/04 (H5N1) | A/WSN/33 (H1N1) | Increase in TNF-α, IP-10 and IFN-β expression in primary human macrophages | No change in M mRNA levels in primary human macrophages | No change in viral growth in primary human macrophages | N/A |
| Ocana-Macchi et al. [15] | 3P | A/chicken/Yamaguchi/7/04 (H5N1) HPAIV | A/swine/Belzig/2/01 (H1N1) | Increase in IFN type I expression in porcine DCs | No change in M1 RNA production | No change in NP expression in porcine DCs | N/A |
| Liniger et al. [2] | 3P | A/duck/Hokkaido/Vac-1/04 (H5N1) LPAIV | A/chicken/Yamaguchi/7/04 (H5N1) HPAIV | Increase in type I IFN production in HD-11 cells | N/A | No change in viral titres in HD-11 cells | N/A |
| Vigeveno et al. [13] | 3P | A/Chicken/Netherlands/emc-3/2014 (H5N8) LPIAV | A/Eurasian Wigeon/Netherlands/4/2016 (H5N8) HPIAV | Increase in IFN-β expression in DEF and DLH cells | Increased activity in HEK293T and QT6 cells | Increase in viral titres in DEF cells | N/A |

^a^ – Contain PA mutations which also affect PA-X residues.

**Supplementary Table 2.** Mutations in the Influenza A virus polymerase and their effect on innate immune responses, polymerase activity, viral growth and virulence.

| Publication | Segment | Mutation | Background virus | Immune response in comparison to background virus | Polymerase activity in comparison to background virus | Viral growth in comparison to background virus | Virulence in animal models in comparison to background  virus |
| --- | --- | --- | --- | --- | --- | --- | --- |
| Graef et al. [16] | PB2 | N9D | A/WSN/33 (H1N1) | Decreased IFN-β production in murine lungs, increased IFN-β production in A549 cells | Decreased transcription and replication in A549 cells | Decreased viral titres in murine lungs, no change in viral replication in MDBK and Vero cells | Decreased virulence (lower mortality and less weight loss) |
| Du et al. [17] | PB2 | N9D | A/WSN/33 (H1N1) | Increased expression of IFN-β and ISG54 and increased nuclear translocation of IRF3 in A549 cells | No change in polymerase activity in 293T cells | No change in viral replication in A549 cells | N/A |
| Te Velthuis et al. [18] | PB2 | N9D | A/WSN/33 (H1N1) (polymerase only) | Increased IFN-β promoter activity in RNP reconstitution in 293T cells | N/A | N/A | N/A |
| Te Velthuis et al. [18] | PB2 | N9D + M81T | A/WSN/33 (H1N1) (polymerase only) | Increased IFN-β promoter activity in RNP reconstitution in 293T cells | N/A | N/A | N/A |
| Te Velthuis et al. [18] | PB2 | N9D + T64M | A/WSN/33 (H1N1) (polymerase only) | Increased IFN-β promoter activity in RNP reconstitution in 293T cells | N/A | N/A | N/A |
| Du et al. [17] | PB2 | Q75H | A/WSN/33 (H1N1) | Increased expression of IFN-β and ISG54 and increased nuclear translocation of IRF3 in A549 cells | No change in polymerase activity in 293T cells | No change in viral replication in A549 cells | N/A |
| Du et al. [17] | PB2 | T76A | A/WSN/33 (H1N1) | Increased expression of IFN-β and ISG54 and increased nuclear translocation of IRF3 in A549 cells | No change in polymerase activity in 293T cells | No change in viral replication in A549 cells | N/A |
| Te Velthuis et al. [18] | PB2 | M81T | A/WSN/33 (H1N1)  (polymerase only) | Increased IFN-β promoter activity in RNP reconstitution in 293T cells | N/A | N/A | N/A |
| Vasilijevic et al. [19] | PB2 | A221T | A/H1N1/California/04/2009 | Increase in ISG56 and Mx production in A549 cells | No change in activity in HEK293T cells | No change in viral titres in A549 cells | Decrease in virulence (lower mortality) in mice |
| Aydillo et al. [20] | PB2 | V249A | A/Puerto Rico/8/1934 (H1N1) with NS1 of HL18NL11 | Decrease in IFN-β expression and IRF3 phosphorylation in A549 cells, decrease in IFN-β expression in murine lungs | Decrease in activity in 293T cells* | No change in viral replication, increased viral titres in the murine lungs | Increased virulence in mice (increased weight loss and mortality) |
| Mok et al. [21] | PB2 | D253N | A/Quail/Hong Kong/G1/1997 (H9N2) | No change in TNF-α production in primary human macrophages | Increased activity in 293T cells* | Increased replication in MDCK cells at 12h post-infection, no change at 6h, 24h and 48h post-infection | N/A |
| Mok et al. [22] | PB2 | T271A | A/Shanghai/2/2013 (H7N9) with PB2-K627E | Increased expression of proinflammatory cytokines | Increased activity in 293T cells | Increased virus replication in murine lungs | No change in virulence (no weight loss) |
| Chen et al. [23] | PB2 | M283I | A/mallard/Huadong/S/2005 (H5N1) | Decrease in expression of IL-6, IFN-β and Mx1 in murine lungs | Decreased activity in 293T cells | Decreased viral titres in the murine lungs | Decreased virulence (improved survival) in mice |
| Chen et al. [23] | PB2 | M283L | A/mallard/Huadong/S/2005 (H5N1) | Increase in expression of IL-6, IFN-β and Mx1 in murine lungs | Increased activity in 293T cells | Increased viral titres in the murine lungs and other organs | Increased virulence (more weight loss, higher mortality and pathological damage) in mice |
| Chen et al. [24] | PB2 | I283M + K526R | A/duck/Eastern China/JY/2014 (H5N8) | Increased expression of a number of immune-related genes, such as IFN-β, IFN-γ, TNF-α, IL6, CXCL10, MX1, IL-1β, DHX58 in murine lungs | N/A | N/A | N/A |
| Gao et al. [25] | PB2 | I292V | A/chicken/Shandong/lx1023/2007 (H9N2) | Decreased expression of IFN-β in A549 cells | Increased activity in 293T cells, but not DF1 cells | Increase in viral titres in A549 and MDCK cells and in murine lungs | Increase in virulence (more weight loss and mortality) |
| Ghorbani et al. [26] | PB2 | D309N | A/turkey/OR/71 (H7N3) | Increased production of biologically active IFN in chicken embryo kidney cells and increased IFN-β mRNA expression in WT-35 cells | N/A | No difference in plaque sizes on chicken embryo kidney cell monolayers | N/A |
| DesRochers et al. [27] | PB2 | E358V | A/shorebird/Delaware/22/2006 (H7N3) | Decreased levels of IL-1α, IL-1β, IL10, IL-12p40 and G-CSF, CCL2, CCL3, KC and GM-CSF in murine lungs | Decreased activity in 293T cells | Decrease in titres at 2 and 9 days post-infection, no change at 3 and 6 days post-infection in murine lungs | Decreased virulence (improved survival and less weight loss) |
| Aydillo et al. [20] | PB2 | I503V | A/Puerto Rico/8/34 (H1N1) with NS1 of HL17NL10 | Decrease in IFN-β expression and IRF3 phosphorylation in A549 cells, decrease in IFN-β expression in murine lungs* | Decrease in activity in 293T cells* | Increase in viral replication in MDCK cells. Increased viral titres in the murine lungs | Increased virulence in mice (increased weight loss and mortality) |
| Zhao et al. [28] | PB2 | T588I | A/Mexico/4486/ 2009 (H1N1) | Decrease in IFN-β expression in A549 cells and murine lungs | Increased activity in 293T cells | Increased viral replication in MDCK, A549 and PK15 cells, increased titres in murine lungs | Increased virulence (higher mortality, more weight loss and more severe lung pathology) in mice |
| Mok et al. [22] | PB2 | Q591K | A/Shanghai/2/2013 (H7N9) with PB2-K627E | Increased expression of IP-10, MCP-3, MCP-1, CXCL-1/KC, MIP-1α and RANTES in murine lungs at 3 or 5 days post-infection | Increased activity in 293T cells | Increased virus replication in murine lungs | Increased virulence (more weight loss) |
| Wang et al. [29] | PB2 | Q591K | A/Quail/Hong Kong/G1/97 (H9N2) | Increased expression of IP-10, MCP-3 and MCP-1 in murine lungs. No difference in MIP-1α, MIP-1β, TNF-α and RANTES expression. Higher influx of neutrophils in the lungs. | Increased polymerase activity in HEK293T cells | Increased replication in NHBE cells | Increased virulence (more weight loss) in mice |
| Li et al. [30] | PB2 | Q591K | A/duck/JX/3286/2009 (H7N9) | Increased levels of RANTES, MCP-1, MCP-3, IFN-α, MIP-1α , MIP-1β, TNF-α and IP-10 in murine lungs | Increased activity in HEK293T cells | Increased replication in A549 cells and murine lungs | No change in pathogenicity in mice |
| Mok et al. [21] | PB2 | Q591K | A/Quail/Hong Kong/G1/1997 (H9N2) | No change in TNF-α production in primary human macrophages | Increased activity in 293T cells* | Increased replication in MDCK cells at 12h post-infection, no change at 6h, 24h and 48h post-infection | N/A |
| Mok et al. [21] | PB2 | Q591K + D253N | A/Quail/Hong Kong/G1/1997 (H9N2) | Increased production of TNF-α and IFN-β in primary human macrophages, and of TNF-α, MCP-1 and MIP-1 in murine lungs | Increased activity in 293T cells | Increased replication in murine lungs and nasal turbinates | Increased virulence (more weight loss) |
| Shinya et al. [31] | PB2 | E627K | A/Hong Kong/486/97 (H5N1) | Persistent neutrophil infiltration and delayed lymphocyte recruitment in murine lungs | N/A | Increased viral titres in murine, but not avian cell lines | Increased virulence (reduction in LD_50_ dose, wide extra-respiratory spread) |
| Fornek et al. [32] | PB2 | E627K | A/Hong Kong/486/97 (H5N1) | Increased and sustained inflammatory responses (IFN signalling and NK cell cytotoxicity), impaired T-cell receptor activation in murine lungs | N/A | Increased viral titres in lungs and dissemination into spleen and brain | Increased virulence (decreased survival and extrapulmonary spread) |
| Tian et al. [33] | PB2 | E627K | A/chicken/Guangdong/V/2008 (H9N2) | Increase in IL-6 and lL-1β production 1-3 days post-infection, and a decrease 5-7 days post-infection. Increase in inflammatory cells and reduction in T-cell infiltration in the murine lungs. | N/A | Increased lung titres | Increased virulence (more weight loss) |
| Yu et al. [34] | PB2 | E627K | A/chicken/Guangdong/ V/2008 (H9N2) | Increased expression of NLRP3, lL-1β and TNF-α in murine lungs and brain | N/A | N/A | N/A |
| Wang et al. [29] | PB2 | E627K | A/Quail/Hong Kong/G1/97 (H9N2) | Increased expression of IP-10, MCP-3 and MCP-1 in murine lungs. No difference in MIP-1α, MIP-1β, TNF-α and RANTES expression. Higher influx of neutrophils in the lungs. | Increased polymerase activity in HEK293T cells | Increased replication in NHBE cells and murine lungs | Increased virulence (more weight loss) in mice |
| Li et al. [30] | PB2 | E627K | A/duck/JX/3286/2009 (H7N9) | Increased expression of TNF-α, IP-10, MCP-1 and MIP-1α in murine lungs | Increased activity in HEK293T cells | Increased replication in A549 cells and murine lungs | Increased pathogenicity in mice (more weight loss) |
| Mok et al. [21] | PB2 | E627K | A/Quail/Hong Kong/G1/1997 (H9N2) | No change in TNF-α production in primary human macrophages | Increased activity in 293T cells* | N/A | N/A |
| Shinya et al. [31] | PB2 | K627E | A/Hong Kong/ 483/97 (H5N1) | Transient neutrophil infiltration and extensive lymphocyte recruitment in murine lungs. | N/A | Decreased viral titres in murine, but not avian cell lines | Decreased virulence (increase in LD_50_ dose, limited extra-respiratory spread) |
| Mok et al. [1] | PB2 | K627E | A/Vietnam/1203/04 (H5N1) | Decrease in IP-10 and IFN-β transcription in pneumocytes; decrease in TNF-α, IP-10 and IFN-β expression in primary human macrophages | N/A | No change in M mRNA levels | N/A |
| Tian et al. [33] | PB2 | K627E | A/chicken/ Guangdong/Ts/2004 (H9N2) | Decrease in IL-6 and lL-1β production. Decrease in infiltration of inflammatory cells and increase in T cell counts in the murine lungs | N/A | Decreased lung titres | Decreased virulence (less weight loss) |
| Mok et al. [22] | PB2 | K627E | A/Shanghai/2/2013 (H7N9) | Decreased expression of proinflammatory cytokines | Decreased activity in 293T cells | Decreased virus replication in murine lungs | Decreased virulence (less weight loss) |
| Weber et al. [35] | PB2 | K627E | A/WSN/33 (H1N1) | Increased binding between RNP and RIG-I and increased RIG-I activation; no difference in IFN-β expression in A549 cells | Decreased activity in HEK293 cells | Decreased replication in HEK293 cells | N/A |
| Wei, Liu [36] | PB2 | K627E | A/Vietnam/1203/2004 (H5N1) | No change in expression of genes involved in DAMPs, NLRs, RLRs and TLRs signalling pathways in Calu-3 cells | N/A | N/A | N/A |
| Zhou et al. [37] | PB2 | D701N | A/New York/1682/2009 (H1N1) | Decrease in IFN-λ and no change in CCL5, IL-6 or IL-8 production in primary alveolar epithelial cells | Increased activity in 293 cells | Increased titres in CMT-93 and human alveolar cells | Increased virulence (higher mortality and more weight loss) |
| Mok et al. [22] | PB2 | D701N | A/Shanghai/2/2013 (H7N9) with PB2-K627E | Increased expression of pro-inflammatory cytokines (IP-10, MCP-3, MCP-1, CXCL-1/KC, MIP-1α, RANTES) in murine lungs at 5 days post-infection | Increased activity in 293T cells | Increased virus replication in murine lungs | Increased virulence (more weight loss) |
| Li et al. [30] | PB2 | D701N | A/duck/JX/3286/2009 (H7N9) | Increased levels of pro-inflammatory cytokines (TNF-α, IP-10, MCP-1, MIP-1α, RANTES, MCP-3, IFN-α, MIP-1β and GM-CSF) in murine lungs | Increased activity in HEK293T cells | Increased replication in A549 cells and murine lungs | No change in virulence in mice (no difference in weight loss) |
| Russell et al. [38] | PB1 | D27N | A/WSN/33 (H1N1) | Increased IFN-β expression in A549 cells | Decreased activity in 293T cells | N/A | N/A |
| Te Velthuis et al. [18] | PB1 | V43I | A/Brevig Mission/1/1918 (H1N1) (polymerase only) | Decreased IFN-β promoter activity in RNP reconstitution in 293T cells | N/A | N/A | N/A |
| Te Velthuis et al. [18] | PB1 | V43I | A/Vietnam/1203/04 (H5N1) | Decreased expression of innate immune genes in A549 cells | N/A | N/A | N/A |
| Russell et al. [38] | PB1 | T677A | A/WSN/33 (H1N1) | Increased IFN-β expression in A549 cells | Increased activity in 293T cells | N/A | N/A |
| Hu et al. [39] | PA | D101G | A/Goose/Jiangsu/k0403/2010 (H5N1) | Increased expression of antiviral cytokines, some pro-inflammatory cytokines and PRR in lungs, spleen and brain of ducks | Increased activity in 293T cells | Increased viral replication in the lungs and brain of mallard ducks | Increased virulence (higher mortality) in mallard ducks |
| DesRochers et al. [27] | PA | P190S | A/shorebird/Delaware/22/2006 (H7N3) | Decreased levels of CCL2, CCL3, KC, and GM-CSF in murine lungs | Decreased activity in 293T cells | Increase in titres at 2 days post-infection, no change at 3, 6, and 9 days post-infection in murine lungs | Decreased virulence (improved survival and less weight loss) |
| Hu et al. [39] | PA | K237E | A/Goose/Jiangsu/k0403/2010 (H5N1) | Increased expression of antiviral cytokines, some pro-inflammatory cytokines and PRR in lungs, spleen and brain of ducks | Increased activity in 293T cells | Increased viral replication in the lungs and brain of mallard ducks | Increased virulence (higher mortality) in mallard ducks |
| Hu et al. [39] | PA | K237E  D101G | A/Goose/Jiangsu/k0403/2010 (H5N1) | Increased expression of antiviral cytokines, some pro-inflammatory cytokines and PRR in lungs, spleen and brain of ducks | No change in activity in 293T cells | Increased viral replication in the lungs and brain of mallard ducks | Increased virulence (higher mortality) in mallard ducks |
| Hu et al. [9] | PA | R353I | A/Chicken/Jiangsu/k0402/2010 (H5N1) | Decreased expression of IL-6, Mx1 CXCL10 and CXCL11 in murine lungs* | Decreased activity in 293T cells | Decreased viral titres in murine lungs* | Decreased virulence (less extrapulmonary spread)* |
| Hu et al. [9] | PA | I353R | A/Goose/Jiangsu/k0403/2010 (H5N1) | Increased expression of IL-6, Mx1 CXCL10 and CXCL11 in murine lungs* | Increased activity in 293T cells | Increased viral titres in murine lungs* | Increased virulence (more extrapulmonary spread)* |
| Fleming-Canepa et al. [40] | PA | T515A | A/Vietnam 1203/2004 (H5N1) | Decreased expression of RIG-I, IFN-β and ISGs in duck lungs and decreased expression of RIG-I in the spleen at 1 day post-infection | N/A | No significant change (slight decrease) in viral replication in duck lungs, decreased replication in spleen at 1 day post-infection | No change in virulence (tissue pathology) in ducks |
| Vasilijevic et al. [19] | PA | D529N | A/H1N1/California/04/2009 | No change in ISG56 and Mx production in A549 cells | No change in activity in HEK293T cells | No change in viral titres in A549 cells | Increase in virulence (higher mortality) with 10^5^ PFU infection dose, no change in virulence with 10^6^ PFU infection dose in mice |
| Vasilijevic et al. [19] | PA + PB2 | PA- D529N  PB2- A221T | A/H1N1/California/04/2009 | No change in ISG56 and Mx production in A549 cells | No change in activity in HEK293T cells | No change in viral titres in A549 cells | Increase in virulence (higher mortality) with 10^5^ PFU infection dose, no change in virulence with 10^6^ PFU infection dose in mice |
| Liedmann et al. [41] | PB1 + PA | PB1-D398E  PB1-R563I | A/Hamburg/04/2009 (H1N1) | Decrease in STAT phosphorylation and IFN-β expression in A549 cells, decrease in IFN expression in murine lungs | No change in activity in A549 cells | Increased viral titres in murine lungs | Increased virulence (higher mortality) in mice |
| Liedmann et al. [41] | PB1 + PA | PB1-D398G PB1-S524G PA-E351K | A/Thailand/1(KAN-1)/2004 (H5N1) | Increase in STAT phosphorylation in A549 cells | N/A | N/A | N/A |
| Liedmann et al. [41] | PB1 + PA | PB1-D398G PB1-S524G PA-E351K | A/Seal/Massachusetts/1/80 (H7N7) | Increase in STAT phosphorylation in A549 cells | N/A | N/A | N/A |
| Liedmann et al. [41] | PB1 + PA | PB1-E398G PB1-S524G  PB1-I563R PA-E351K | A/Puerto-Rico/8/34 (H1N1) | Increase in STAT phosphorylation and IFN-β expression in A549 and NHBE cells, increased IRF3 activation in A549 cells, increased in IFN-β expression in murine lungs | No change in activity in A549 cells | Decreased viral titres in the murine lungs | Decreased virulence (lower mortality and weight loss) in mice |
| Liedmann et al. [14] | PB1 +PA | PB1-E398G PB1-S524G  PB1-I563R PA-E351K | A/PR/8/34 (H1N1) | Increased STAT1 phosphorylation in A549 cells | N/A | N/A | N/A |

* no statistical testing

# References

1. Mok KP, Wong CHK, Cheung CY, Chan MC, Lee SMY, Nicholls JM, Guan Y, Peiris JSM (2009) Viral genetic determinants of H5N1 influenza viruses that contribute to cytokine dysregulation. The Journal of infectious diseases 200 (7):1104-1112. doi:<https://dx.doi.org/10.1086/605606>

2. Liniger M, Moulin HR, Sakoda Y, Ruggli N, Summerfield A (2012) Highly pathogenic avian influenza virus H5N1 controls type I IFN induction in chicken macrophage HD-11 cells: a polygenic trait that involves NS1 and the polymerase complex. Virology journal 9:7. doi:<https://dx.doi.org/10.1186/1743-422X-9-7>

3. Watanabe C, Uchida Y, Ito H, Ito T, Saito T (2011) Host immune-related gene responses against highly pathogenic avian influenza virus infection in vitro differ among chicken cell lines established from different organs. Veterinary immunology and immunopathology 144 (3-4):187-199. doi:<https://dx.doi.org/10.1016/j.vetimm.2011.10.002>

4. Forero A, Tisoncik-Go J, Watanabe T, Zhong G, Hatta M, Tchitchek N, Selinger C, Chang J, Barker K, Morrison J, Berndt JD, Moon RT, Josset L, Kawaoka Y, Katze MG (2015) The 1918 Influenza Virus PB2 Protein Enhances Virulence through the Disruption of Inflammatory and Wnt-Mediated Signaling in Mice. Journal of virology 90 (5):2240-2253. doi:<https://dx.doi.org/10.1128/JVI.02974-15>

5. Watanabe T, Zhong G, Russell CA, Nakajima N, Hatta M, Hanson A, McBride R, Burke DF, Takahashi K, Fukuyama S, Tomita Y, Maher EA, Watanabe S, Imai M, Neumann G, Hasegawa H, Paulson JC, Smith DJ, Kawaoka Y (2014) Circulating avian influenza viruses closely related to the 1918 virus have pandemic potential. Cell Host Microbe 15 (6):692-705. doi:<https://doi.org/10.1016/j.chom.2014.05.006>

6. Williams GD, Pinto AK, Doll B, Boon ACM (2016) A North American H7N3 Influenza Virus Supports Reassortment with 2009 Pandemic H1N1 and Induces Disease in Mice without Prior Adaptation. Journal of virology 90 (9):4796-4806. doi:<https://dx.doi.org/10.1128/JVI.02761-15>

7. Park S-J, Kim E-H, Kwon H-I, Song M-S, Kim SM, Kim Y-I, Si Y-J, Lee I-W, Nguyen HD, Shin OS, Kim C-J, Choi YK (2018) Altered virulence of Highly Pathogenic Avian Influenza (HPAI) H5N8 reassortant viruses in mammalian models. Virulence 9 (1):133-148. doi:<https://dx.doi.org/10.1080/21505594.2017.1366408>

8. Li OTW, Chan MCW, Leung CSW, Chan RWY, Guan Y, Nicholls JM, Poon LLM (2009) Full factorial analysis of mammalian and avian influenza polymerase subunits suggests a role of an efficient polymerase for virus adaptation. PloS one 4 (5):e5658. doi:<https://dx.doi.org/10.1371/journal.pone.0005658>

9. Hu J, Hu Z, Song Q, Gu M, Liu X, Wang X, Hu S, Chen C, Liu H, Liu W, Chen S, Peng D, Liu X (2013) The PA-gene-mediated lethal dissemination and excessive innate immune response contribute to the high virulence of H5N1 avian influenza virus in mice. Journal of virology 87 (5):2660-2672. doi:<https://dx.doi.org/10.1128/JVI.02891-12>

10. Wang Q, Zhang S, Jiang H, Wang J, Weng L, Mao Y, Sekiguchi S, Yasui F, Kohara M, Buchy P, Deubel V, Xu K, Sun B, Toyoda T (2012) PA from an H5N1 highly pathogenic avian influenza virus activates viral transcription and replication and induces apoptosis and interferon expression at an early stage of infection. Virology journal 9:106. doi:<https://dx.doi.org/10.1186/1743-422X-9-106>

11. Sakabe S, Takano R, Nagamura-Inoue T, Yamashita N, Nidom CA, Quynh Le M, Iwatsuki-Horimoto K, Kawaoka Y (2013) Differences in cytokine production in human macrophages and in virulence in mice are attributable to the acidic polymerase protein of highly pathogenic influenza A virus subtype H5N1. J Infect Dis 207 (2):262-271. doi:<https://doi.org/10.1093/infdis/jis523>

12. Dlugolenski D, Jones L, Howerth E, Wentworth D, Tompkins SM, Tripp RA (2015) Swine Influenza Virus PA and Neuraminidase Gene Reassortment into Human H1N1 Influenza Virus Is Associated with an Altered Pathogenic Phenotype Linked to Increased MIP-2 Expression. Journal of virology 89 (10):5651-5667. doi:<https://dx.doi.org/10.1128/JVI.00087-15>

13. Vigeveno RM, Poen MJ, Parker E, Holwerda M, de Haan K, van Montfort T, Lewis NS, Russell CA, Fouchier RAM, de Jong MD, Eggink D (2020) Outbreak severity of highly pathogenic avian influenza A(H5N8) viruses is inversely correlated to polymerase complex activity and interferon induction. Journal of virology. doi:<https://dx.doi.org/10.1128/JVI.00375-20>

14. Liedmann S, Hrincius ER, Anhlan D, McCullers JA, Ludwig S, Ehrhardt C (2014) New virulence determinants contribute to the enhanced immune response and reduced virulence of an influenza A virus A/PR8/34 variant. The Journal of infectious diseases 209 (4):532-541. doi:<https://dx.doi.org/10.1093/infdis/jit463>

15. Ocana-Macchi M, Ricklin ME, Python S, Monika G-A, Stech J, Stech O, Summerfield A (2012) Avian influenza A virus PB2 promotes interferon type I inducing properties of a swine strain in porcine dendritic cells. Virology 427 (1):1-9. doi:<https://dx.doi.org/10.1016/j.virol.2012.01.037>

16. Graef KM, Vreede FT, Lau Y-F, McCall AW, Carr SM, Subbarao K, Fodor E (2010) The PB2 subunit of the influenza virus RNA polymerase affects virulence by interacting with the mitochondrial antiviral signaling protein and inhibiting expression of beta interferon. Journal of virology 84 (17):8433-8445. doi:<https://dx.doi.org/10.1128/JVI.00879-10>

17. Du Y, Xin L, Shi Y, Zhang TH, Wu NC, Dai L, Gong D, Brar G, Shu S, Luo J, Reiley W, Tseng YW, Bai H, Wu TT, Wang J, Shu Y, Sun R (2018) Genome-wide identification of interferon-sensitive mutations enables influenza vaccine design. Science 359 (6373):290-296. doi:<https://doi.org/10.1126/science.aan8806>

18. Te Velthuis AJW, Long JC, Bauer DLV, Fan RLY, Yen H-L, Sharps J, Siegers JY, Killip MJ, French H, Oliva-Martin MJ, Randall RE, de Wit E, van Riel D, Poon LLM, Fodor E (2018) Mini viral RNAs act as innate immune agonists during influenza virus infection. Nature microbiology 3 (11):1234-1242. doi:<https://dx.doi.org/10.1038/s41564-018-0240-5>

19. Vasilijevic J, Zamarreno N, Oliveros JC, Rodriguez-Frandsen A, Gomez G, Rodriguez G, Perez-Ruiz M, Rey S, Barba I, Pozo F, Casas I, Nieto A, Falcon A (2017) Reduced accumulation of defective viral genomes contributes to severe outcome in influenza virus infected patients. PLoS Pathog 13 (10):e1006650. doi:<https://doi.org/10.1371/journal.ppat.1006650>

20. Aydillo T, Ayllon J, Pavlisin A, Martinez-Romero C, Tripathi S, Mena I, Moreira-Soto A, Vicente-Santos A, Corrales-Aguilar E, Schwemmle M, Garcia-Sastre A (2018) Specific Mutations in the PB2 Protein of Influenza A Virus Compensate for the Lack of Efficient Interferon Antagonism of the NS1 Protein of Bat Influenza A-Like Viruses. Journal of virology 92 (7). doi:<https://dx.doi.org/10.1128/JVI.02021-17>

21. Mok CK, Yen HL, Yu MY, Yuen KM, Sia SF, Chan MC, Qin G, Tu WW, Peiris JS (2011) Amino acid residues 253 and 591 of the PB2 protein of avian influenza virus A H9N2 contribute to mammalian pathogenesis. J Virol 85 (18):9641-9645. doi:<https://doi.org/10.1128/JVI.00702-11>

22. Mok CKP, Lee HHY, Lestra M, Nicholls JM, Chan MCW, Sia SF, Zhu H, Poon LLM, Guan Y, Peiris JSM (2014) Amino acid substitutions in polymerase basic protein 2 gene contribute to the pathogenicity of the novel A/H7N9 influenza virus in mammalian hosts. Journal of virology 88 (6):3568-3576. doi:<https://dx.doi.org/10.1128/JVI.02740-13>

23. Chen S, Xie Y, Su X, Xue J, Wang X, Du Y, Qin T, Peng D, Liu X (2020) Substitutions in the PB2 methionine 283 residue affect H5 subtype avian influenza virus virulence. Transboundary and emerging diseases 67 (6):2554-2563. doi:<https://dx.doi.org/10.1111/tbed.13601>

24. Chen S, Wang X, Su X, Miao X, Qin T, Peng D, Liu X (2021) Deep sequencing of the transcriptome from murine lung infected with H5N8 subtype avian influenza virus with combined substitutions I283M and K526R in PB2 gene. Infection, genetics and evolution : journal of molecular epidemiology and evolutionary genetics in infectious diseases 87:104672. doi:<https://dx.doi.org/10.1016/j.meegid.2020.104672>

25. Gao W, Zu Z, Liu J, Song J, Wang X, Wang C, Liu L, Tong Q, Wang M, Sun H, Sun Y, Liu J, Chang K-C, Pu J (2019) Prevailing I292V PB2 mutation in avian influenza H9N2 virus increases viral polymerase function and attenuates IFN-beta induction in human cells. The Journal of general virology 100 (9):1273-1281. doi:<https://dx.doi.org/10.1099/jgv.0.001294>

26. Ghorbani A, Abundo MC, Ji H, Taylor KJM, Ngunjiri JM, Lee C-W (2020) Viral Subpopulation Screening Guides in Designing a High Interferon-Inducing Live Attenuated Influenza Vaccine by Targeting Rare Mutations in NS1 and PB2 Proteins. Journal of virology 95 (2). doi:<https://dx.doi.org/10.1128/JVI.01722-20>

27. DesRochers BL, Chen RE, Gounder AP, Pinto AK, Bricker T, Linton CN, Rogers CD, Williams GD, Webby RJ, Boon AC (2016) Residues in the PB2 and PA genes contribute to the pathogenicity of avian H7N3 influenza A virus in DBA/2 mice. Virology 494:89-99. doi:<https://doi.org/10.1016/j.virol.2016.04.013>

28. Zhao Z, Yi C, Zhao L, Wang S, Zhou L, Hu Y, Zou W, Chen H, Jin M (2014) PB2-588I enhances 2009 H1N1 pandemic influenza virus virulence by increasing viral replication and exacerbating PB2 inhibition of beta interferon expression. Journal of virology 88 (4):2260-2267. doi:<https://dx.doi.org/10.1128/JVI.03024-13>

29. Wang C, Lee HHY, Yang ZF, Mok CKP, Zhang Z (2016) PB2-Q591K Mutation Determines the Pathogenicity of Avian H9N2 Influenza Viruses for Mammalian Species. PloS one 11 (9):e0162163. doi:<https://dx.doi.org/10.1371/journal.pone.0162163>

30. Li W, Lee HHY, Li RF, Zhu HM, Yi G, Peiris JSM, Yang ZF, Mok CKP (2017) The PB2 mutation with lysine at 627 enhances the pathogenicity of avian influenza (H7N9) virus which belongs to a non-zoonotic lineage. Scientific reports 7 (1):2352. doi:<https://dx.doi.org/10.1038/s41598-017-02598-z>

31. Shinya K, Hamm S, Hatta M, Ito H, Ito T, Kawaoka Y (2004) PB2 amino acid at position 627 affects replicative efficiency, but not cell tropism, of Hong Kong H5N1 influenza A viruses in mice. Virology 320 (2):258-266. doi:<https://doi.org/10.1016/j.virol.2003.11.030>

32. Fornek JL, Gillim-Ross L, Santos C, Carter V, Ward JM, Cheng LI, Proll S, Katze MG, Subbarao K (2009) A single-amino-acid substitution in a polymerase protein of an H5N1 influenza virus is associated with systemic infection and impaired T-cell activation in mice. Journal of virology 83 (21):11102-11115. doi:<https://dx.doi.org/10.1128/JVI.00994-09>

33. Tian J, Qi W, Li X, He J, Jiao P, Zhang C, Liu G-Q, Liao M (2012) A single E627K mutation in the PB2 protein of H9N2 avian influenza virus increases virulence by inducing higher glucocorticoids (GCs) level. PloS one 7 (6):e38233. doi:<https://dx.doi.org/10.1371/journal.pone.0038233>

34. Yu M, Zhang K, Qi W, Huang Z, Ye J, Ma Y, Liao M, Ning Z (2014) Expression pattern of NLRP3 and its related cytokines in the lung and brain of avian influenza virus H9N2 infected BALB/c mice. Virology journal 11:229. doi:<https://dx.doi.org/10.1186/s12985-014-0229-5>

35. Weber M, Sediri H, Felgenhauer U, Binzen I, Banfer S, Jacob R, Brunotte L, Garcia-Sastre A, Schmid-Burgk JL, Schmidt T, Hornung V, Kochs G, Schwemmle M, Klenk H-D, Weber F (2015) Influenza virus adaptation PB2-627K modulates nucleocapsid inhibition by the pathogen sensor RIG-I. Cell host & microbe 17 (3):309-319. doi:<https://dx.doi.org/10.1016/j.chom.2015.01.005>

36. Wei K, Liu X (2015) Phylogenetic Analysis and Functional Characterization of the Influenza A H5N1 PB2 Gene. Transboundary and emerging diseases 64 (2):374-388. doi:<https://dx.doi.org/10.1111/tbed.12376>

37. Zhou B, Pearce MB, Li Y, Wang J, Mason RJ, Tumpey TM, Wentworth DE (2013) Asparagine substitution at PB2 residue 701 enhances the replication, pathogenicity, and transmission of the 2009 pandemic H1N1 influenza A virus. PloS one 8 (6):e67616. doi:<https://dx.doi.org/10.1371/journal.pone.0067616>

38. Russell AB, Elshina E, Kowalsky JR, Te Velthuis AJW, Bloom JD (2019) Single-Cell Virus Sequencing of Influenza Infections That Trigger Innate Immunity. J Virol 93 (14). doi:<https://doi.org/10.1128/JVI.00500-19>

39. Hu J, Hu Z, Mo Y, Wu Q, Cui Z, Duan Z, Huang J, Chen H, Chen Y, Gu M, Wang X, Hu S, Liu H, Liu W, Liu X, Liu X (2013) The PA and HA gene-mediated high viral load and intense innate immune response in the brain contribute to the high pathogenicity of H5N1 avian influenza virus in mallard ducks. Journal of virology 87 (20):11063-11075. doi:<https://dx.doi.org/10.1128/JVI.00760-13>

40. Fleming-Canepa X, Aldridge JR, Jr., Canniff L, Kobewka M, Jax E, Webster RG, Magor KE (2019) Duck innate immune responses to high and low pathogenicity H5 avian influenza viruses. Veterinary microbiology 228:101-111. doi:<https://dx.doi.org/10.1016/j.vetmic.2018.11.018>

41. Liedmann S, Hrincius ER, Guy C, Anhlan D, Dierkes R, Carter R, Wu G, Staeheli P, Green DR, Wolff T, McCullers JA, Ludwig S, Ehrhardt C (2014) Viral suppressors of the RIG-I-mediated interferon response are pre-packaged in influenza virions. Nature communications 5:5645. doi:<https://dx.doi.org/10.1038/ncomms6645>
